# Flip bifurcation analysis and mathematical modeling of cholera disease by taking control measures

**DOI:** 10.1038/s41598-024-59640-0

**Published:** 2024-05-13

**Authors:** Aqeel Ahmad, Fakher Abbas, Muhammad Farman, Evren Hincal, Abdul Ghaffar, Ali Akgül, Murad Khan Hassani

**Affiliations:** 1 Department of Mathematics, Ghazi University D G Khan, Dera Ghazi Khan, 32200 Pakistan; 2 Department of Computer Science and Mathematics, Lebanese American University, Beirut, Lebanon; 3 Mathematics Research Center, Near East University, Near East Boulevard, 99138 Nicosia, North Cyprus Cyprus; 4 Department of Mathematics, Near East University, Near East Boulevard, 99138 Nicosia, North Cyprus Cyprus; 5 Department of Mathematics, Art and Science Faculty, Siirt University, 56100 Siirt, Turkey; 6 Department of Mathematics, Ghazni University, Ghazni, Afghanistan

**Keywords:** Mathematical modeling, Boundedness, Non-local kernel, Uniqueness, Flip bifurcation, Atangana–Toufik, Applied mathematics, Computational science

## Abstract

To study the dynamical system, it is necessary to formulate the mathematical model to understand the dynamics of various diseases which are spread in the world wide. The objective of the research study is to assess the early diagnosis and treatment of cholera virus by implementing remedial methods with and without the use of drugs. A mathematical model is built with the hypothesis of strengthening the immune system, and a ABC operator is employed to turn the model into a fractional-order model. A newly developed system SEIBR, which is examined both qualitatively and quantitatively to determine its stable position as well as the verification of flip bifurcation has been made for developed system. The local stability of this model has been explored concerning limited observations, a fundamental aspect of epidemic models. We have derived the reproductive number using next generation method, denoted as “$R_{0}$”, to analyze its impact rate across various sub-compartments, which serves as a critical determinant of its community-wide transmission rate. The sensitivity analysis has been verified according to its each parameters to identify that how much rate of change of parameters are sensitive. Atangana–Toufik scheme is employed to find the solution for the developed system using different fractional values which is advanced tool for reliable bounded solution. Also the error analysis has been made for developed scheme. Simulations have been made to see the real behavior and effects of cholera disease with early detection and treatment by implementing remedial methods without the use of drugs in the community. Also identify the real situation the spread of cholera disease after implementing remedial methods with and without the use of drugs. Such type of investigation will be useful to investigate the spread of virus as well as helpful in developing control strategies from our justified outcomes.

## Introduction

Biology has involved mathematics since Fibonacci employed the well-known Fibonacci series to explain population expansion in the early twelfth century^1^. Daniel Bernoulli described the effect of small shapes applied a mathematics on small shapes The term bio math was first used by Johannes Ranke in 1901^2^. Bio math is primarily theoretical analysis of mathematical models to analyze rules that made the structure development as well as behavior system^3^. It is made for comprehending curiosities of bio organisms. Mathematics has principality subsidize and further advancement in natural sciences, it may play the key role in living organisms of biological sciences^4^. That is why it is accepted that essential parts to bring the student at early stages by that time, a new comer with only the basic fundamental knowledge to the associative aspect of mathematical biology^5^. In mathematical biology, a case study can be divided into many stages^6^. Phase one is a representation of biological method which can raise more biological quires in which mathematic might be proved beneficial for giving solution. Second phase is to explain a mathematical process which can describe a proper biological model. The next phase is to implement mathematical models as well as additional process, such that the model may be used to create mathematical rules. The last phase is to conclude the mathematical outcomes for given topic in the light of biological methods. Fractional calculus (FC)^7^, and its applications are a powerful tool for interpreting various problems in various fields of engineering and science. There are several descriptions of integrated and derivative operators such as Riemann Liouville, Granwald Letnikov, Caputo^8^, Caputo and Fabrizio (CF)^9^, Atangana Baleanu^10^. By the bacterial species Vibrio cholera an noxious intestinal disease caused is Cholera^11^. Infective bacterial germs are tired in vomit or fecal of infected persons and escalation in the infected persons by the faeces-oral avenue^12^. When a man devour take deteriorated food or drink water then serious diarrhea and enormous the vomiting is by this disease^13^. This disease is for several years have been a main cause of hygienics heed and also an gesture of the insufficiency of social conveniences in is developed countries. In recent the disease cholera flare-up in Tanzania^14^ Zimbabwe^15^,Kenya^16^ Ethiopia^17^, Yemen^18^, and other nations keep the disease on the worldwide public health agenda. They carry on passage of cholera associate certain cooperation of germs, climate, and the persons^19^, which is direct person-to-person and indirect climate to person the way to go. Due the immense Cholera has been broadly researched for its effects on public health and economic development Theoretically and clinically, cholera can be annihilate through applicable measures such as treatment of infected persons, and hygienic practices. Realizing the, effort have been made to developed workable, prevention and interventions the planning over years.

Numerous mathematicians have created and also monitored mathematical models to monitored the dynamics and control of spreadable disease (see,^20–24^). An attempts has been made to understand the intricate mechanism of cholera transmission^25^. In^26^, through the application of Pontryagin’s maximum principle, a mathematical model of cholera outbreaks with an ideal control system was created and examined. Researchers concentrated on vaccinations, medical treatment, and educational initiatives as a means to regulate the widespread outbreak of cholera among populations. However, the model’s control parameters did not incorporate a safe household water supply. Citing the authors^27^, created a model in Zimbabwe from 2008 to 2009 for the purpose to investigate the cholera spread. The direct (person-to-person) as well as indirect (climate-to-person) transit pathways are incorporated in that model, and cholera outbreaks in Africa ensure the vitality of the person-to-person transportation route. Research by^28^ altered the suggested cholera model, while^29^ examined the best interruption tactics and additional control possibilities, nonetheless, no one considered the risk of infection from humans. Adapted and examined a deterministic cholera model in Tanzania. Integrated the water treatment and control systems with the human educational trips. However, they did not conduct a quantitative evaluation of the beginning reproducing number that has an initiation value for the disease transmission in the research evaluation of this model. Cholera disease mathematical model was also proposed by the authors in^30^, where by public health is the primary control strategy for cholera. Yet, that model have not include protected domestic water storage as control. Many Other new models of strategic cholera include those that^31–35^, the ability to control cholera using appropriate preservation techniques. However, most of these models do not take into consideration personal data, hygienic guidelines, adjunctive care for afflicted individuals, control systems, or campaigns. Recently, academics have begun researching the use of fractional-order derivatives for enhanced mathematical models. Numerous partial and nonlocal derivatives have been proposed by a lot of researchers. Full order derivatives are devalued; see^22,36,37^. For instance, Caputo considerably refined fractional order differential theory subsequent to Riemann–Liouville’s introduction of the concept^38,39^.

In^40^, the dynamics of smoking behavior under the influence of educational and media programs is modeled in the presence of two control strategies. In^41^, they extend a Susceptible Lock down Infective Recovered Environmental Reservoir (SLIRV) model of novel corona virus with effect of lock-down to optimal control problem and they used Hamilton and Lagrangian formulations to investigate the existence of optimum control. The model was analyzed by stability theory of differential equations and computer simulation^42^. In^43^, they had formulated and analyzed a mathematical model to investigate the impacts of lock-down on the dynamics of forestry biomass, wildlife species and pollution. Authors introduced an enhanced version of model with incorporating minimum interest rates and maximum investment demands in^44^. In^45^, they addressed the solution of a Caputo fractional-order Black-Scholes model using an analytic method named the modified initial guess homotopy perturbation method. Lassa fever is a viral infection that is most common in West Africa and presented a fractional order model of Lassa disease^46^. In^47^ proposed a fractional order mathematical model to analyze the impact of high risk quarantine and vaccination on COVID-19 transmission. Authors attempted to investigated the spread and control of the COVID-19 virus in Nigeria using the Caputo fractional order derivative in a proposed model^48^.

Both Caputo and Riemann–Liouville operators are a partial derivative with known as kernels^49^. Modeling physical issues applying fractional order derivatives, it has received considerable attention in both engineering and biological systems, see^50–56^. This is by reason of Fractional order operators go on the stability section, capturing the genetic and gesture properties of memory which occur in the both engineering and biological programs^57^. Every one knows this integers can not obtain order derivatives. Spatial systems and memory effects are simple without the influence of external forces^58^. Also, partial order derivatives do bring better fits to actual data for various disease models , see^39,59,60^. Integers do not involves through fractional operators in the advantages of physical modeling issues. Fractional Order derivatives induced by the this studies on fractional-order cholera disease model and recommended transference that absorb the effect of individuals information sanitation practices, expedition and the treatment of infected individuals. Here the previous model is given in^12^:

1 $$\begin{aligned} \begin{aligned} D S(t) =&\varpropto ^q-(1-\vartheta ^{q})\beta ^{q}_{h}S(t)I(t) -(1- w^{q}_{2})\beta ^{q}_{e} \frac{S(t)B(t)}{k^q+ B(t)}- (\nu ^q+\vartheta ^q)S(t) \\ D I(t) =&(1-\vartheta ^{q})\beta ^{q}_{h}S(t) I(t)-(1-w^{q}_{2})\beta ^{q}_{e}\frac{S(t)B(t)}{k^q+B(t)}-(\nu ^q+d^q+w_1^q\alpha ^q)I(t) \\ D B(t) =&\varsigma ^q I(t)-(w_2^q +\zeta ^q )B(t) \\ D R(t) =&w_1^q\alpha ^qI(t)+\vartheta ^qS(t)-\nu ^qR(t) \end{aligned} \end{aligned}$$

Initial conditions corresponds to the aforementioned system:

$$ S^{0} = S( 0 ) $$ , $$ B^{0} = B( 0 ) $$ , $$ I^{0} = I( 0 ) $$ and $$ R^{0} = R( 0 ) $$ .

### Definition 1.1

The Liouville–Caputo sense (ABC) in the form of fractional order derivative of Atangana–Baleanu is given by^10^

2 $$\begin{aligned} {}^{ABC}_x D^{\tau }_{t} z(t) = \frac{AB(\tau )}{n-\tau } \int _{x}^{t} \frac{d^n}{dw^n} z(w) E_\tau \left[ -\tau \frac{(t-w)^\tau }{n-\tau } \right] dw, \ \ \ \ \ \ \ \ \ \ \ \ \ \ n-1< \tau < n, \end{aligned}$$

where $E_\tau$ and $AB(\tau )$ are the Mittag-Leffler function and normalization function, respectively with $AB(0)=AB(1)=1$.

By using transform of laplace of (2), we get

3 $$\begin{aligned} {[}{}^{ABC}_x D^{\tau }_{t} \{z(t)\}]s = \frac{AB(\tau )}{1-\tau } \frac{s^{\tau }(s) L[z(t)] - z ( 0 ) s^{\tau - 1} }{ \tau \frac{1}{1-\tau } + s^\tau }. \end{aligned}$$

The sumudu transform (ST) of (2) is given by

4 $$\begin{aligned} ST[{}^{ABC}_x D^{\tau }_{t} \{z(t)\}](s) = \frac{B(\tau )}{1-\tau } \left( \tau \Gamma (\tau +1)E_\tau \left( -\frac{1}{1-\tau }v^\tau \right) \right) \times [ST(z(t))-z(0)] \end{aligned}$$

For order $\tau$, the fractional integral of Atangana–Baleanu of a function *z*(*t*) is obtained by

5 $$\begin{aligned} {}^{ABC}_x I^{\tau }_{t} \{z(t)\} = \frac{(1 - \tau ) z ( t )}{B-\tau } + \frac{\tau }{ B(\tau ) \Gamma (\tau )} \int _{x}^{t} z ( s )( t - s )^{\tau - 1}ds. \end{aligned}$$

## Formulation of SEIBR model

**Figure 1:**
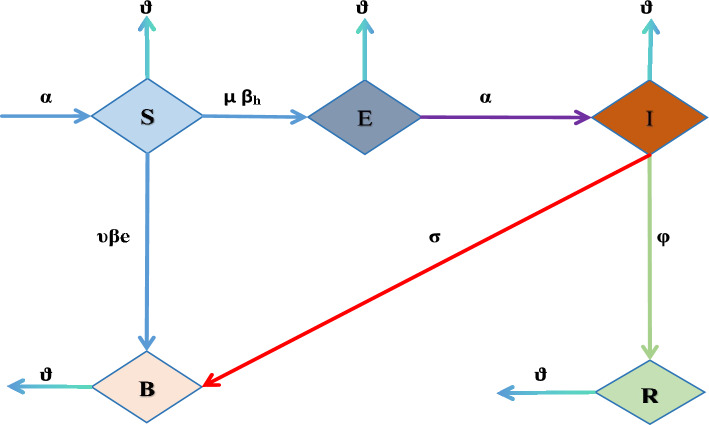
Flowchart of Cholera disease model.

Here for the spread of cholera disease we used differential equations model which has Fractal fractional-order derivative. The sections of the study that are recommended here are utilized to draw attention to the Vibrio bacteria’s concentration in the environment as well as each person’s physical appearance status. Flowchart of cholera disease model is given by Fig. 1. The suggested and examined mathematical model is supervised by these supposition:

1. For humans, we split the segment into four subclasses: Susceptible individuals (S (t)), exposed people (E(t)), infectious people $\{\text {I(t)}\}$, and recovered people $\{\text {R ( t )}\}$. N(t) therefore represents the total number of humans, which is defined as: $N = R + I + E + S, B ( t )$ denote the focus of Vibrio’s in the environment, and parameters are denoted by *K*, that are shows the halved-intensity of Vibrio’s in the climate.
2. The entire document, parameters and variables both are assumed the non-negative and are defined as follows: $\varpropto$ and $\nu$ mean to show new enrollment rate and natural fatalities rate of human being, that is represent the middling time in which peoples spend in the period of infectious.
3. People are thought to become aware of the disease through education and publicity, and they are connected to the rate of “recovered class” individuals who have not lost their immunity or awareness during the cholera outbreak. Those who are infected but fail to take care of themselves eventually perish from the disease at a rate of “$\vartheta$” and after receiving effective medical treatment, they recover from the disease at an average of “$\phi$”.
4. Vibrio bacterium concentration and enlargement, the parameter ‘$\mu$’ indicates the rate of recovery for infected individuals, ‘$\sigma$’ indicates the spread of infected human flow vibrio’s in climate, and $'\nu '$ indicates the spread of exposed human flow vibrio bacteria in climate. Parameters ‘$\beta _{e}$’, ‘$\beta _{h}$’ represent the forces of infectious among the environment-to-persons being, person-to-person transportation.

As a consequence, the system of differential equations shown below can explain a host-vector epidemic model with nonlinear incidence

6 $$\begin{aligned} \begin{aligned} D S(t) =&\varpropto - \beta _{h} \mu E(t) S(t) - \beta _{e} \nu B(t) S(t) - \vartheta S(t) \\ D E(t) =&\beta _{h} \mu E(t) S(t) - \left( \alpha + \vartheta \right) E(t) \\ D I(t) =&\alpha E(t) - \left( \phi + \vartheta \right) I(t) \\ D B(t) =&\sigma I(t) + \beta _{e} \nu B(t) S(t) - \vartheta B(t) \\ D R(t) =&\phi I(t) - \vartheta R(t) \\ \end{aligned} \end{aligned}$$

started to under the following conditions:

$$ \,S^0 = \,S(0),$$ $$ \,E^0 = \,E(0),$$ $$ I^0 = I(0) $$, $$ B^0 = B(0) $$, and $$ \,R^0 = \,R(\,0) $$ .

The previously provided model becomes, by employing the Atangana–Baleanu in caputo sense ( ABC ) fractional Operator:

7 $$\begin{aligned} \begin{aligned} {}_{\xi }^{ABC}D_t^\xi S(t) =&\varpropto - \beta _{h} \mu \,E(\,t) \,S(\,t) - \beta _{e} \nu \,B(\,t) \,S(\,t) - \vartheta \,S(\,t) \\ {}_{\xi }^{ABC}D_t^\xi E(t) =&\beta _{h} \mu E(t) S(\,t) - \left( \alpha + \vartheta \right) E(\,t) \\ {}_{\xi }^{ABC}D_t^\xi I(t) =&\alpha E(\,t) - \left( \phi + \vartheta \right) I(\,t) \\ {}_{\xi }^{ABC}D_t^\xi B(t) =&\sigma I(\,t) + \beta _{e} \nu B(\,t) S(\,t) - \vartheta B(\,t) \\ {}_{\xi }^{ABC}D_t^\xi R(t) =&\phi I(\,t) - \vartheta R(\,t) \\ \end{aligned} \end{aligned}$$

Here ’${}^{ABC}_{\xi }D^{\xi }_{t}$ ’ indicates the fractional derivative of the Atangana–Baleanu in caputo sense ( ABC ) fractional Operator, and $1> \xi > 0$. Initial circumstances related to the system 7 are: $\,S^0 = \,S(0),$ $B^0 = B(0)$, $\,E^0 = \,E(\,0) ,$ $\,I^0 = \,I(\,0)$ and $\,R^0 = \,R(\,0)$ .

To approximate the solution of the system (7), we utilize the Sumudu transform operator provided in (8).The aforementioned are the ways that the operator is utilized by both sides of the system: (7):

8 $$\begin{aligned} \frac{B(\xi )\xi \Gamma (\xi +1)}{1-\xi } E_\xi \left( \frac{- \,w^\xi }{\,1-\,\xi }\right) \left[ ST S (\,t) - \,S(\,0) \right]= & {} ST \left[ \varpropto - \beta _{h} \mu E(\,t) S(\,t) - \beta _{e} \nu B(\,t) S(\,t) - \vartheta S(\,t) \right] \nonumber ,\\ \frac{B(\xi )\xi \Gamma (\xi +1)}{1-\xi } E_\xi \left( \frac{\,-w^\xi }{\,1-\,\xi }\right) \left[ ST E (\,t)-E(\,0) \right]= & {} ST \left[ \beta _{h} \mu E(\,t) S(\,t) -(\alpha + \vartheta ) E(\,t) \right] ,\nonumber \\ \frac{B(\xi )\xi \Gamma (\xi +1)}{1-\xi } E_\xi \left( \frac{\,-w^\xi }{\,1-\,\xi }\right) \left[ ST I(\,t)-I(\,0) \right]= & {} ST \left[ \alpha E(\,t) -( \phi + \vartheta ) I(\,t)\right] , \nonumber \\ \frac{B(\xi )\xi \Gamma (\xi +1)}{1-\xi } E_\xi \left( \frac{\,-w^\xi }{\,1-\,\xi }\right) \left[ ST B(\,t)-B(\,0) \right]= & {} ST \left[ \sigma I(\,t)+(\beta _{e} \nu S(\,t) - \vartheta )B(\,t)\right] , \nonumber \\ \frac{B(\xi )\xi \Gamma (\xi +1)}{1-\xi } E_\xi \left( \frac{\,-w^\xi }{\,1-\,\xi }\right) \left[ ST R(\,t)-R(\,0) \right]= & {} ST \left[ \phi I(\,t) - \vartheta R(\,t)\right] . \end{aligned}$$

By rearrangement system (8), we possess.

9 $$\begin{aligned} ST[S ( t )]= & {} \frac{1-\xi }{B(\xi )\xi \Gamma (\xi +1)E_\xi }\left( -\frac{1-\xi }{w^\xi }\right) \times ST\left[ \varpropto - \beta _{h} \mu E(t) S(t) - \beta _{e} \nu B(t) S(t) - \vartheta S(t) \right] + S(0),\nonumber \\ ST[E ( t )]= & {} \frac{1-\xi }{B(\xi )\xi \Gamma (\xi +1)E_\xi }\left( -\frac{1-\xi }{w^\xi }\right) \times ST\left[ \beta _{h} \mu E(t) S(t) -(\alpha + \vartheta ) E(t) \right] + E(0), \nonumber \\ ST[E ( t )]= & {} \frac{1-\xi }{B(\xi )\xi \Gamma (\xi +1)E_\xi }\left( -\frac{1-\xi }{w^\xi }\right) \times ST\left[ \alpha E(t) -( \phi + \vartheta ) I(t)\right] + I(0), \nonumber \\ ST[B ( t )]= & {} \frac{1-\xi }{B(\xi )\xi \Gamma (\xi +1)E_\xi }\left( -\frac{1-\xi }{w^\xi }\right) \times ST\left[ \sigma I(t) + ( \beta _{e} \nu S(t) - \vartheta ) B(t)\right] + B(0), \nonumber \\ ST[R ( t )]= & {} \frac{1-\xi }{B(\xi )\xi \Gamma (\xi +1)E_\xi }\left( -\frac{1-\xi }{w^\xi }\right) \times ST\left[ \phi I(t) - \vartheta R(t)\right] + R(0). \end{aligned}$$

On the both sides of the equations system (9) by utilizing the inverse sumudu transform, then the system of equations result:

$$\begin{aligned} S(t)= & {} (ST)^{-1}\left[ \frac{1-\xi }{B(\xi )\xi \Gamma (\xi +1)E_\xi }\left( -\frac{1-\xi }{w^\xi }\right) \times ST\left\{ \varpropto - \beta _{h} \mu E(t) S(t) - \beta _{e} \nu B(t) S(t) - \vartheta S(t) \right\} \right] + S(0), \\ E(t)= & {} (ST)^{-1}\left[ \frac{1-\xi }{B(\xi )\xi \Gamma (\xi +1)E_\xi }\left( -\frac{1-\xi }{w^\xi }\right) \times ST\left\{ \beta _{h} \mu E(t) S(t) -(\alpha + \vartheta ) E(t)\right\} \right] + E(0), \\ I(t)= & {} (ST)^{-1}\left[ \frac{1-\xi }{B(\xi )\xi \Gamma (\xi +1)E_\xi }\left( -\frac{1-\xi }{w^\xi }\right) \times ST\left\{ \alpha E(t) -( \phi + \vartheta ) I(t)\right\} \right] + I(0), \\ B(t)= & {} (ST)^{-1}\left[ \frac{1-\xi }{B(\xi )\xi \Gamma (\xi +1)E_\xi }\left( -\frac{1-\xi }{w^\xi }\right) \times ST\left\{ \sigma I(t) + ( \beta _{e} \nu B(t)S(t) - \vartheta ) B(t)\right\} \right] + B(0), \\ R(t)= & {} (ST)^{-1}\left[ \frac{1-\xi }{B(\xi )\xi \Gamma (\xi +1)E_\xi }\left( -\frac{1-\xi }{w^\xi }\right) \times ST\left\{ \phi I(t) - \vartheta R(t)\right\} \right] + R(0). \end{aligned}$$

As a result, the following is acquired:

$$\begin{aligned} (S)_{k+1}(t)= & {} (ST)^{-1}\Bigg [\frac{1-\xi }{B(\xi )\xi \Gamma (\xi +1)E_\xi }\left( -\frac{1-\xi }{w^\xi }\right) \times ST\{\varpropto - \beta _{h} \mu E(t) S(t) - \beta _{e} \nu B(t) S(t) - \vartheta S(t) \}\Bigg ] + (S)_k(0), \\ (E)_{k+1}(t)= & {} (ST)^{-1}\Bigg [\frac{1-\xi }{B(\xi )\xi \Gamma (\xi +1)E_\xi }\left( -\frac{1-\xi }{w^\xi }\right) \times ST \{\beta _{h} \mu E(t) S(t) -(\alpha + \vartheta ) E(t)\} \Bigg ] + (E)_k(0), \\ (I)_{k+1}(t)= & {} (ST)^{-1}\left[ \frac{1-\xi }{B(\xi )\xi \Gamma (\xi +1)E_\xi }\left( -\frac{1-\xi }{w^\xi }\right) \times ST \{ \alpha E(t) -(\phi + \vartheta ) I(t)\} \right] + (I)_k(0), \\ (B)_{k+1}(t)= & {} (ST)^{-1}\left[ \frac{1-\xi }{B(\xi )\xi \Gamma (\xi +1)E_\xi }\left( -\frac{1-\xi }{w^\xi }\right) \times ST \{ \sigma I(t) + (\beta _{e} \nu B(t)S(t) - \vartheta ) B(t)\} \right] + (B)_k(0), \\ (R)_{k+1}(t)= & {} (ST)^{-1}\left[ \frac{1-\xi }{B(\xi )\xi \Gamma (\xi +1)E_\xi }\left( -\frac{1-\xi }{w^\xi }\right) \times ST \{ \phi I(t) - \vartheta R(t)\} \right] + (R)_k(0). \end{aligned}$$

And the obtained solution of equation (7) is presented as:

$$ S(t) = \lim _{k\rightarrow \infty } (S)_k(t); E(t) = \lim _{k\rightarrow \infty } (E)_k(t); I(t) = \lim _{k\rightarrow \infty } (I)_k(t); B(t) = \lim _{k\rightarrow \infty } (B)_k(t); R(t) = \lim _{k\rightarrow \infty } (R)_k(t). $$

Suppose there is a Bananch Space $( S, |\cdot |)$. and a self map of *S* is *J*. consider $(B)_{k+1} = ( g )(J(B)_k)$ represent a particular iterative process. For $(B)_{k+1} = J(B)_k$ the following requirements need to be met.

1. In the set of fixed point *J* at least the one element is maintained.
2. $(B)_k$ approaches a fixed point. $Q \in F(J)$
3. $$\lim _{k\rightarrow \infty } (S)_k(t) = Q$$

### Theorem 1

Let there is a Bananch Space $( S, |\cdot |)$. And a self map of *S* is *J* satisfying:

10 $$\begin{aligned} \Vert J_{(S)} - J_{(B)} \Vert \le \theta \Vert S - J_{(S)} \Vert + \theta \Vert S - B \Vert , \end{aligned}$$

for all $S, B \in S$, where $1> \theta > 0.$ Suppose *J* represent a Picard $J-$stable.

Consider above Eq., we get.

11 $$\begin{aligned} \frac{1-\xi }{B(\xi )\xi \Gamma (\xi +1)E_\xi \left( -\frac{1}{1-\xi }w^\xi \right) }, \end{aligned}$$

The fractional lagrange multiplier is connected to the aforementioned equation.

### Proof

Given *J*, define it as a self map given by it.

12 $$\begin{aligned} J[(S)_{k+1}]= & {} [(S)_{k+1}]\nonumber \\= & {} (S)_k + (ST)^{-1}\Bigg \{\frac{1-\xi }{B(\xi )\xi \Gamma (\xi +1)E_\xi \left( -\frac{1}{1-\xi }w^\xi \right) } \times ST[\varpropto - \mu \beta _{h} S_k E_k - \nu \beta _{e} S_k B_k - \vartheta S_k]\Bigg \}, \nonumber \\ J[(E)_{k+1}]= & {} [(E)_{k+1}] = (E)_k + (ST)^{-1}\Bigg \{\frac{1-\xi }{B(\xi )\xi \Gamma (\xi +1)E_\xi \left( -\frac{1}{1-\xi }w^\xi \right) } \times ST[\mu \beta _{h} S_k E_k - \alpha E_k - \vartheta E_k]\Bigg \},\nonumber \\ J[(I)_{k+1}]= & {} [(I)_{k+1}] = (I)_k + (ST)^{-1}\Bigg \{\frac{1-\xi }{B(\xi )\xi \Gamma (\xi +1)E_\xi \left( -\frac{1}{1-\xi }w^\xi \right) } \times ST[\alpha E_k - (\phi + \vartheta ) I_k]\Bigg \},\nonumber \\ J[(B)_{k+1}]= & {} [(B)_{k+1}] = (B)_k + (ST)^{-1}\Bigg \{ \frac{1-\xi }{B(\xi )\xi \Gamma (\xi +1)E_\xi \left( -\frac{1}{1-\xi }w^\xi \right) } \times ST[\sigma I_k + \nu \beta _{e} S_k B_k - \vartheta B_k]\Bigg \}, \nonumber \\ J[(R)_{k+1}]= & {} [(R)_{k+1}] = (R)_k + (ST)^{-1}\Bigg \{ \frac{1-\xi }{B(\xi )\xi \Gamma (\xi +1)E_\xi \left( -\frac{1}{1-\xi }w^\xi \right) } \times ST[\phi I_k - \vartheta R_k]\Bigg \}. \end{aligned}$$

Utilizing the norm’s characteristics and keeping the triangle inequality in mind, we obtain

13 $$\begin{aligned} \Vert J[(S)_{k}] - J[(S)_{m}]\Vert\le & {} \Vert (S)_{k} - (S)_{m}\Vert + (ST)^{-1}\Bigg \{ \frac{1-\xi }{B(\xi ) \xi \Gamma (\xi +1)E_\xi \left( -\frac{1}{1-\xi }w^\xi \right) } \nonumber \\{} & {} \times ST[\varpropto - \mu \beta _{h}(S_kE_k- S_m E_m) - \nu \beta _{e} (S_k B_k - S_m B_m) - \vartheta (S_k - S_m)]\Bigg \}, \nonumber \\ \Vert J[(E)_{k}]-J[(E)_{m}]\Vert\le & {} \Vert (E)_k - (E)_m\Vert + (ST)^{-1}\Bigg \{ \frac{1-\xi }{B(\xi )\xi \Gamma (\xi +1)E_\xi \left( -\frac{1}{1-\xi }w^\xi \right) } \nonumber \\{} & {} \times ST[\mu \beta _{h} (S_k E_k - S_m E_m)- \alpha (E_k - E_m) - \vartheta (E_k - E_m)]\Bigg \}, \nonumber \\ \Vert J[(I)_{k}]-J[(I)_{m}]\Vert\le & {} \Vert (I)_k-(I)_m\Vert + (ST)^{-1}\Bigg \{ \frac{1-\xi }{B(\xi )\xi \Gamma (\xi +1)E_\xi \left( -\frac{1}{1-\xi }w^\xi \right) } \nonumber \\{} & {} \times ST[\alpha (E_k - E_m) - \phi (I_k - I_m) - \vartheta (I_k - I_m)]\Bigg \}, \nonumber \\ \Vert J[(B)_{k}]- J[(B)_{m}]\Vert\le & {} \Vert (B)_k - (B)_m\Vert + (ST)^{-1}\Bigg \{ \frac{1-\xi }{B(\xi )\xi \Gamma (\xi +1)E_\xi \left( -\frac{1}{1-\xi }w^\xi \right) } \nonumber \\{} & {} \times ST[\sigma (I_k - I_m) + \nu \beta _{e} (S_k B_k - S_mB_m) - \vartheta (B_k -B_k )]\Bigg \}, \nonumber \\ \Vert J[(R)_{k}]-J[(R)_{m}]\Vert\le & {} \Vert (R)_k-(R)_m\Vert + (ST)^{-1}\Bigg \{ \frac{1-\xi }{B(\xi )\xi \Gamma (\xi +1)E_\xi \left( -\frac{1}{1-\xi }w^\xi \right) } \nonumber \\{} & {} \times ST[\phi (I_k - I_m) - \vartheta (R_k -R_m )]\Bigg \}. \end{aligned}$$

*J* satisfies the requirements mentioned in Theorem 1. Therefore *J* must be Picard *J*-stable.

14 $$\begin{aligned} \theta= & {} (0,0,0,0,0)\nonumber \\ \theta= & {} \left\{ \begin{array}{ll} \Vert (S)_k - (S)_m\Vert \times \Vert (S)_m - (S)_k\Vert + \varpropto - \beta _{h} \mu \Vert E_k S_k - E_m S_m \Vert &{} \\ - \beta _{e} \nu \Vert B_k S_k - B_m S_m\Vert - \vartheta \Vert (S_k - S_m)\Vert &{} \\ \times \Vert (E)_k - (E)_m\Vert \times \Vert (E)_m - (E)_k\Vert + \beta _{h} \mu \Vert E_k S_k - E_m S_m \Vert - \alpha \\ (E_k - E_m)\Vert - \vartheta \Vert (E_k - E_m)\Vert &{}\\ \times \Vert (I)_k - (I)_m\Vert \times \Vert (I)_m - (I)_k\Vert + \alpha \Vert (E_k - E_m)\Vert &{}\\ - \phi \Vert (I_k - I_m)\Vert - \vartheta \Vert (I_k - I_m)\Vert &{} \\ \times \Vert (B)_k - (B)_m\Vert \times \Vert (B)_m - (B)_k\Vert + \sigma \Vert (I_k - I_m)\Vert + &{}\\ \nu \beta _{e} \Vert (S_k B_k - S_m B_m)\Vert - \vartheta \Vert (B_k - B_m)\Vert &{} \\ \times \Vert (R)_n - (R)_m\Vert \times \Vert (R)_m - (R)_n\Vert + \phi \Vert (I_k - I_m)\Vert - \vartheta \Vert (R_k -R_m)\Vert . \end{array} \right. \end{aligned}$$

and we add that *J* is Picard $J-$stable. $\square$

### Theorem 2

The particular solution of the Eq. 7 using the iteration method is unique singular solution.

### Proof

Consider the Hilbert Space $J= K^2((i,j)\times (0,p))$ that follows.

15 $$\begin{aligned} j: (i,j) \times \left[ 0, \textrm{T} \right] \rightarrow {\mathbb {R}}, \ \ \ \ \ \ \ \int \int ijhdidj < \infty \end{aligned}$$

The following operators are taken into consideration in this.

16 $$\begin{aligned} \theta= & {} (0,0,0,0,0)\nonumber \\ \theta= & {} \left\{ \begin{array}{ll} \varpropto - \mu \beta _{h} S E - \nu \beta _{e} S B - \vartheta S; &{} \\ \mu \beta _{h} S E - \alpha E - \vartheta E; &{} \\ \alpha E - \phi I - \vartheta I; &{} \\ \sigma I + \nu \beta _{e} S B - \vartheta B; &{} \\ \phi I - \vartheta R. &{} \end{array} \right. \end{aligned}$$

We verify by the inner product

$$\begin{aligned}{} & {} \textrm{T}\left[ \{(\,S)_{\,11} - (\,S)_{\,12}\}; \{(\,E)_{\,21} - (\,E)_{\,22}\}; \{(\,I)_{\,31} - (\,I)_{\,32}\}; \{(\,B)_{\,41} - (\,B)_{\,42}\};\right. \\ {}{} & {} \left. \quad \{\,(R)_{\,51} - (\,R)_{\,52}\}; (u_1, u_2, u_3, u_4, u_5) \right] . \end{aligned}$$

In cases whereas $((\,S)_{\,11} - (\,S)_{\,12});$ $((\,E)_{\,21} - (E)_{22};$ $((\,I)_{\,31} - (\,I)_{\,32});$ $((\,B)_{\,41} - (B)_{42});$ $((\,R)_{\,51} - (\,R)_{\,52});$ consist of the system’s particular solutions. Considering the internal workings and standard, may have.

$$\begin{aligned}{} & {} \Big [ \varpropto - \mu \beta _{h} \{(S)_{11} - (S)_{12}\} \{(E)_{21} - (E)_{22}\} - \nu \beta _{e} \{(S)_{11} - (S)_{12}\} \{(B)_{41} - (B)_{42}\} - \vartheta \{(S)_{11} - (S)_{12}\} \Big ] \\{} & {} \quad \le \Big [ \varpropto \Vert u_1\Vert - \mu \beta _{h} \Vert (S)_{11} - (S)_{12}\Vert \Vert (E)_{21} - (E)_{22}\Vert \Vert u_1\Vert - \nu \beta _{e} \Vert (S)_{11} - (S)_{12}\Vert \Vert (B)_{41} - (B)_{42}\Vert \Vert u_1\Vert \\{} & {} \qquad - \vartheta \Vert (S)_{11} - (S)_{12}\Vert \Vert u_1\Vert \Big ]; \\{} & {} \Big [\mu \beta _{h} \{(S)_{11} - (S)_{12}\} \{(\,E)_{\,21} - (\,E)_{\,22}\} - \alpha \{(\,E)_{\,21} - (\,E)_{\,22}\} - \vartheta \{(\,E)_{\,21} - (\,E)_{22}\} \Big ] \\ {}{} & {} \quad \le \Big [\mu \beta _{h} \Vert (S)_{11} - (S)_{12}\Vert \Vert (E)_{21} - (E)_{22}\Vert \Vert u_2\Vert - \alpha \Vert (E)_{21} - (E)_{22}\Vert \Vert u_2\Vert - \vartheta \Vert (E)_{21} - (E)_{22}\Vert \Vert u_2\Vert \Big ]; \\ {}{} & {} \Big [\alpha \{(E)_{21} - (E)_{22}\} - \phi \{(I)_{31} - (I)_{32}\} - \vartheta \{(I)_{31} - (I)_{32}\} \Big ] \\ {}{} & {} \quad \le \Big [\alpha \Vert (E)_{21} - (E)_{22}\Vert \Vert u_3\Vert - \phi \Vert (I)_{31} - (I)_{32}\Vert \Vert u_3\Vert - \vartheta \Vert (I)_{31} - (I)_{32}\Vert \Vert u_3\Vert \Big ]; \\ {}{} & {} \Big [\sigma \{(I)_{31} - (I)_{32}\} + \nu \beta _{e} \{(S)_{11} - (S)_{12}\} \{(B)_{41} - (B)_{42}\} - \vartheta \{(B)_{41} - (B)_{42}\} \Big ] \\ {}{} & {} \quad \le \Big [\sigma \Vert (I)_{31} - (I)_{32}\Vert \Vert u_4\Vert + \nu \beta _{e} \Vert (S)_{11} - (S)_{12}\Vert \Vert (B)_{41} - (B)_{42}\Vert \Vert u_4\Vert - \vartheta \Vert (B)_{41} - (B)_{42}\Vert \Vert u_4\Vert \Big ]; \\ {}{} & {} \Big [\phi \{(I)_{31} - (I)_{32}\} - \vartheta \{(R)_{51} - (R)_{52}\}\Big ] \\ {}{} & {} \quad \le \Big [\phi \Vert (I)_{31}-(I)_{32}\Vert \Vert u_5\Vert - \vartheta \Vert (R)_{51} - (R)_{52}\Vert \Vert u_5\Vert \Big ]. \end{aligned}$$

Both strategies coincidentally converge to the correct solution for significant amounts $i_1$, $i_2$, $i_3$, $i_4$, and $i_5$ as well. Through the application of the topological concepts, we are able to derive five extremely tiny positive parameters. $(\Upsilon _{i_1}$, $\Upsilon _{i_2}$, $\Upsilon _{i_3}$, $\Upsilon _{i_4}$, and $\Upsilon _{i_5})$.

$$\begin{aligned} \frac{\Upsilon _{i_1}}{\Psi _1}> & {} \Vert S - (S)_{11}\Vert , \Vert S - (S)_{12}\Vert , \\ \frac{\Upsilon _{i_2}}{\Psi _2}> & {} \Vert E - (E)_{21}\Vert , \Vert E - (E)_{22}\Vert , \\ \frac{\Upsilon _{i_3}}{\Psi _3}> & {} \Vert I - (I)_{31}\Vert , \Vert I - (I)_{32}\Vert , \\ \frac{\Upsilon _{i_4}}{\Psi _4}> & {} \Vert B - (B)_{41}\Vert , \Vert B \,-\, (\,B)_{\,42}(\,t)\Vert , \\ \frac{\Upsilon _{i_5}}{\Psi _5}> & {} \Vert R(\,t) \,-\, (\,R)_{\,51}(\,t)\Vert , \Vert R(\,t) \,-\, (\,R)_{\,52}(\,t)\Vert , \end{aligned}$$

where

$$\begin{aligned} \Psi _1= & {} 5\Big [ \varpropto - \mu \beta _{h} \Vert (\,S)_{\,11}(\,t)\,-\,(\,S)_{\,12}(\,t)\Vert \Vert (\,E)_{\,21}(\,t)\,\\{} & {} -\,(\,E)_{\,22}(\,t)\Vert \,-\, \nu \beta _{e} \Vert (\,S)_{\,11}(\,t)\,-\,(\,S)_{\,12}(\,t)\Vert \Vert (\,B)_{\,41}(\,t)\,-\,(\,B)_{\,42}(\,t)\Vert \\{} & {} - \vartheta \Vert (\,S)_{\,11}(\,t)\,-\,(\,S)_{\,12}(\,t)\Vert \Big ] \Vert u_1\Vert ; \\ \Psi _2= & {} 5\Big [\mu \beta _{h} \Vert (\,S)_{\,11}(\,t)\,-\,(\,S)_{\,12}(\,t)\Vert \Vert (\,E)_{\,21}(\,t)\,\\{} & {} -\,(\,E)_{\,22}(\,t)\Vert - \alpha \Vert (\,E)_{\,21}(\,t)\,-\,(\,E)_{\,22}(\,t)\Vert - \vartheta \Vert (\,E)_{\,21}(\,t)\,-\,(\,E)_{\,22}(\,t)\Big ] \Vert u_2\Vert ,\\ \Psi _3= & {} 5\Big [\alpha \Vert (\,E)_{\,21}(\,t)\,-\,(\,E)_{\,22}(\,t)\Vert - \phi \Vert (\,I)_{\,31}(\,t)\,\\{} & {} -\,(\,I)_{\,32}(\,t)\Vert - \vartheta \Vert (I)_{31} - (I)_{32}\Vert \Big ] \Vert u_3\Vert , \\ \Psi _4= & {} 5\Big [\sigma \Vert (I)_{31} - (I)_{32}\Vert + \nu \beta _{e} \Vert (S)_{11} - (S)_{12}\Vert \Vert (B)_{41} \\{} & {} - (B)_{42}\Vert - \vartheta \Vert (B)_{41} - (B)_{42}\Vert \Big ] \Vert u_4\Vert ,\\ \Psi _5= & {} 5\Big [\phi \Vert (I)_{31}-(I)_{32}\Vert - \vartheta \Vert (R)_{51} - (R)_{52}\Vert \Big ]\Vert u_5\Vert ,\\ \end{aligned}$$

where

$$\begin{aligned}{} & {} \Big [ \varpropto - \mu \beta _{h} \Vert (S)_{11}-(S)_{12}\Vert \Vert (E)_{21}-(E)_{22}\Vert - \nu \beta _{e} \Vert (S)_{11} - (S)_{12}\Vert \Vert (B)_{41} - (B)_{42}\Vert - \vartheta \Vert (S)_{11} - (S)_{12}\Vert \Big ] \Vert u_1\Vert \ne 0, \\{} & {} \Big [\mu \beta _{h} \Vert (S)_{11} - (S)_{12}\Vert \Vert (E)_{21} - (E)_{22} \Vert - \alpha \Vert (E)_{21} - (E)_{22}\Vert - \vartheta \Vert (E)_{21}-(E)_{22}\Big ] \Vert u_2\Vert \ne 0,\\{} & {} \Big [\alpha \Vert (E)_{21} - (E)_{22}\Vert - \phi \Vert (I)_{31} - (I)_{32}\Vert - \vartheta \Vert (I)_{31} - (I)_{32}\Vert \Big ] \Vert u_3\Vert \ne 0, \\{} & {} \Big [\sigma \Vert (I)_{31} - (I)_{32}\Vert + \nu \beta _{e} \Vert (S)_{11} - (S)_{12}\Vert \Vert (B)_{41} - (B)_{42}\Vert - \vartheta \Vert (B)_{41} - (B)_{42}\Vert \Big ] \Vert u_4\Vert \ne 0,\\{} & {} \Big [\phi \Vert (I)_{31}-(I)_{32}\Vert - \vartheta \Vert (R)_{51} - (R)_{52}\Vert \Big ]\Vert u_5\Vert \ne 0, \end{aligned}$$

where $$ \Vert \,u_1\Vert \ne 0 $$, $$ \Vert \,u_2 \Vert \, \ne 0 $$, $$ \Vert \,u_3 \Vert \ne 0 $$, $$ \Vert \, u_4 \Vert \ne 0 $$, $$ \Vert \,u_5 \Vert \ne 0 $$; $$ \Vert (S)_{11} - (S)_{12}\Vert = 0 $$, $$ \Vert (E)_{21} - (E)_{22}\Vert = 0 $$, $$ \Vert (I)_{31} - (I)_{32}\Vert = 0 $$, $$ \Vert (B)_{41} - (B)_{42}\Vert = 0 $$, $$ (R)_{51} - (R)_{52}\Vert = 0 $$.

We can write $(S)_{11} = (S)_{12}$, $(B)_{41} = (B)_{42}$, $(E)_{21} = (E)_{22}$, $(I)_{31} = (I)_{32}$, $(R)_{51} = (R)_{52}$.

From above result, we conclude that system has unique singular solution for all sub-compartments. We uses the technique to consider two solutions for each sub-compartment but in the last equation, we verify that both are equal which shows the uniqueness of solutions. $\square$

## Analysis equilibrium points

In the aforementioned portion of the research, pandemic points of equilibrium and disease-free points of equilibrium are two different types of equilibrium points. To discover them, the system’s equations’ right-hand sides are set to be as zero. The disease-free equilibrium points $F_0$ represent the steady state outcome in which there is no cholera infection in the community. Now, by setting ${}_{\xi }^{ABC}D_t^\xi S(t) = {}_{\xi }^{ABC}D_t^\xi E(t) = {}_{\xi }^{ABC}D_t^\xi I(t) = {}_{\xi }^{ABC}D_t^\xi B(t) = {}_{\xi }^{ABC}D_t^\xi R(t)=0$ in system of the Eq. (7), we get

$$\begin{aligned} 0 &=  \varpropto - \mu \beta _{h} E S - \nu \beta _{e} B S - \vartheta S \\  0 &=  \mu \beta _{h} E S - \left( \vartheta + \alpha \right) E \\ 0 &=  \alpha E - (\phi + \vartheta ) I \\ 0 &=  \sigma I + \nu \beta _{e} B S - \vartheta B \\ 0 &=  \phi I - \vartheta R \\ \end{aligned}$$

After simplification, we get

$$\begin{aligned} \varpropto &=  \mu \beta _{h} E S + \nu \beta _{e} B S + \vartheta S \\ \mu \beta _{h} E S &=  \left( \vartheta + \alpha \right) E \\ \alpha E &=  \left( \phi + \vartheta \right) I \\ \sigma I + \nu \beta _{e} B S &=  \vartheta B \\ \phi I &=  \vartheta R \\ \end{aligned}$$

For this model (7), the point of equilibrium without disease is

$$\begin{aligned} F(S, E, I, B, R) = F_{S000R}\Big (\frac{\varpropto }{\vartheta }, 0, 0, 0, 0 \Big ) \end{aligned}$$

as well as the pandemic point of equilibrium as follows $F^*(S^*, E^*, I^*, B^*, R^*)$ where

$$\begin{aligned} S^*= & {} \frac{ \alpha + \vartheta }{\beta _{h}\mu }\\ E^*= & {} -\frac{(\vartheta +\phi ) \left( \alpha \vartheta - \beta _{h} \varpropto \mu +\vartheta ^2\right) (\alpha \beta _{e} \nu - \beta _{h} \vartheta \mu +\beta _{e} \vartheta \nu )}{\beta _{h} \mu (\alpha +\vartheta ) \left( \alpha \beta _{e} \vartheta \nu -\alpha \beta _{e} \nu \sigma +\alpha \beta _{e} \nu \phi -\beta _{h} \vartheta ^2 \mu -\beta _{h} \vartheta \mu \phi +\beta _{e} \vartheta ^2 \nu +\beta _{e} \vartheta \nu \phi \right) }\\ I^*= & {} -\frac{\alpha \left( \alpha \vartheta -\beta _{h} \varpropto \mu +\vartheta ^2\right) (\alpha \beta _{e} \nu -\beta _{h} \vartheta \mu +\beta _{e} \vartheta \nu )}{\beta _{h} \mu (\alpha +\vartheta ) \left( \alpha \beta _{e} \vartheta \nu -\alpha \beta _{e} \nu \sigma +\alpha \beta _{e} \nu \phi - \beta _{h} \vartheta ^2 \mu -\beta _{h} \vartheta \mu \phi + \beta _{e} \vartheta ^2 \nu + \beta _{e} \vartheta \nu \phi \right) } \\ B^*= & {} \frac{\alpha \sigma \left( \alpha \vartheta -\beta _{h} \varpropto \mu +\vartheta ^2\right) }{(\alpha +\vartheta ) \left( \alpha \beta _{e} \vartheta \nu -\alpha \beta _{e} \nu \sigma +\alpha \beta _{e} \nu \phi -\beta _{h} \vartheta ^2 \mu -\beta _{h} \vartheta \mu \phi +\beta _{e} \vartheta ^2 \nu +\beta _{e} \vartheta \nu \phi \right) } \\ R^*= & {} -\frac{\alpha \phi (\alpha \vartheta -\beta \varpropto \mu +\vartheta ^2) (\alpha \beta _{e} \nu -\beta _{h} \vartheta \mu +\beta _{e} \vartheta \nu )}{\beta _{h} \vartheta \mu (\alpha +\vartheta ) \left( \alpha \beta _{e} \vartheta \nu -\alpha \beta _{e} \nu \sigma +\alpha \beta _{e} \nu \phi -\beta _{h} \vartheta ^2 \mu -\beta _{h} \vartheta \mu \phi +\beta _{e} \vartheta ^2 \nu +\beta _{e} \vartheta \nu \phi \right) } \end{aligned}$$

### Local stability analysis of equilibrium points

Here, we talk about theorems and related proofs that characterize the local stability of equilibria.

#### Theorem 3

The local dynamic stability of the disease-free equilibria in the model is guaranteed when $R_0 < \, 1$.

#### Proof

The Jacobian Matrix model is that

17 $$\begin{aligned} J_{(SEIBR)}( S, E, I, B, R )= \left[ \begin{array}{ccccc} -\mu \beta _{h} E &{} \quad - \nu \beta _{e}B - \vartheta &{}\quad -\mu \beta _{h} S &{}\quad - \nu \beta _{e}S &{} 0 \\ - \mu \beta _{h}E &{} \quad \mu \beta _{h}S - \alpha - \vartheta &{} \quad 0 &{}\quad 0 &{}\quad 0 \\ 0 &{} \quad \alpha &{} \quad -\phi - \vartheta &{}\quad 0&{}\quad 0 \\ \beta _e \nu B &{} \quad 0 &{}\quad \sigma &{}\quad \beta _e \nu S &{}\quad 0 \\ 0 &{}\quad 0 &{}\quad \phi &{}\quad 0 &{} \quad \vartheta \\ \end{array} \right] \end{aligned}$$

The Jacobian Matrix model on the equilibria points is that

18 $$\begin{aligned} J_{(SEIBR)}\Big (\frac{\varpropto }{\vartheta }, 0, 0, 0, 0 \Big )= \left[ \begin{array}{ccccc} - \vartheta &{} -\frac{ \mu \beta _{h} \varpropto }{\vartheta } &{}\quad 0 &{}\quad -\frac{ \nu \beta _{e} \varpropto }{\vartheta } &{}\quad 0 \\ 0 &{} \frac{ \mu \beta _{h} \varpropto }{\vartheta } - \alpha - \vartheta &{}\quad 0 &{}\quad 0 &{}\quad 0 \\ 0 &{}\quad \alpha &{}\quad -\phi - \vartheta &{}\quad 0 &{}\quad 0 \\ 0 &{} \quad 0 &{}\quad \sigma &{} \quad \frac{ \nu \beta _{e} \varpropto }{\vartheta } - \vartheta &{}\quad 0 \\ 0 &{} \quad 0 &{} \quad \phi &{} \quad 0 &{} \quad -\vartheta \\ \end{array} \right] \end{aligned}$$

For the characteristic equation $|J_{0} - \lambda I| = 0$

19 $$\begin{aligned} \left| \begin{array}{ccccc} - \vartheta &{} \quad -\frac{ \mu \beta _{h} \varpropto }{\vartheta } &{} \quad 0 &{}\quad -\frac{ \nu \beta _{e} \varpropto }{\vartheta } &{}\quad 0 \\ 0 &{}\quad \frac{ \mu \beta _{h} \varpropto }{\vartheta } - \alpha - \vartheta &{}\quad 0 &{}\quad 0 &{}\quad 0 \\ 0 &{}\quad \alpha &{}\quad -\phi - \vartheta &{}\quad 0 &{} \quad 0 \\ 0 &{} \quad 0 &{}\quad \sigma &{}\quad \frac{ \nu \beta _{e} \varpropto }{\vartheta } - \vartheta &{}\quad 0 \\ 0 &{}\quad 0 &{}\quad \phi &{} \quad 0 &{} \quad -\vartheta \\ \end{array} \right| = 0 \end{aligned}$$

The Characteristic polynomial of (19) is

$$\begin{aligned}{} & {} -\frac{(-\vartheta -\lambda ) (\vartheta + \lambda ) (-\vartheta - \lambda - \phi ) (\beta _{e} \varpropto \nu - \vartheta ^2 - \vartheta \lambda )( - \alpha + \frac{\beta _{h} \varpropto \mu }{\vartheta }-\vartheta -\lambda )}{\vartheta } \end{aligned}$$

The Eigen Values of model is

$$\begin{aligned} \lambda _1 & = -\vartheta ,\\ \lambda _2 & = -\vartheta ,\\ \lambda _3 & =  \frac{-\alpha \vartheta + \beta _{h} \varpropto \mu - \vartheta ^2}{\vartheta },\\ \lambda _4 & =  \frac{\beta _{e} \varpropto \nu - \vartheta ^2}{\vartheta },\\ \lambda _5 & = - \vartheta - \phi . \end{aligned}$$

Since the all the eigen values have negative real parts, then system is locally stable which is required condition for local stability taken from the characteristic equation. $\square$

## Reproduction number and its analysis

The $R_0$ is the average number of new infections caused by an infectious person in the early or late stages of the disease through direct contact with a cholera disease in a population that is solely susceptible. The cholera infection can begin to spread in the population when $R_0 > 1$, but not when $R_0 < 1$. The matrices *F* and *V* are examined at the disease-free equilibrium point $F_0$ and are Jacobian matrices that correspond to the functions *F* and *V*, respectively. The element at the (*i*, *j*) location of matrix *F* in the context of these matrices represents the rate at which an infected individual in compartment *j* transfers the virus to compartment i. The element at location (*i*, *j*) in the matrix *V* denotes the spread of an infection that already exists. Evaluation of the spectral radius of the matrix $FV^{-1}$ at the disease-free equilibrium point is required for the reproduction number computation. This matrix, which is called the Next Generation Matrix, is described as follows:

$$\begin{aligned} J_0= & {} \left( \begin{array}{ccccc} -\vartheta &{} \quad -\frac{\beta _{h} \varpropto \mu }{\vartheta } &{} \quad 0 &{}\quad -\frac{\beta _{e} \varpropto \nu }{\vartheta } &{}\quad 0 \\ 0 &{}\quad \frac{\beta _{h} \varpropto \mu }{\vartheta }-(\alpha +\vartheta ) &{}\quad 0 &{} \quad 0 &{}\quad 0 \\ 0 &{} \quad \alpha &{} \quad -(\vartheta +\phi ) &{}\quad 0 &{} \quad 0 \\ 0 &{} \quad 0 &{} \quad \sigma &{}\quad \frac{\beta _{e} \varpropto \nu }{\vartheta }-\vartheta &{}\quad 0 \\ 0 &{}\quad 0 &{}\quad \phi &{}\quad 0 &{}\quad -\vartheta \\ \end{array} \right) \\ J_0= & {} F-V \end{aligned}$$

The vectors *F* and *V* in our developed model can be found using the equation 

$$\begin{aligned} F= & {} \left( \begin{array}{ccccc} 0 &{}\quad 0 &{} \quad 0 &{} \quad 0 &{} \quad 0 \\ 0 &{} \quad \frac{\beta _{h} \varpropto \mu }{\vartheta } &{} \quad 0 &{} \quad 0 &{} \quad 0 \\ 0 &{} \alpha &{} 0 &{} 0 &{} 0 \\ 0 &{}\quad 0 &{}\quad \sigma &{} \quad \frac{\beta _{e} \varpropto \nu }{\vartheta } &{}\quad 0 \\ 0 &{}\quad 0 &{} \quad \phi &{} \quad 0 &{}\quad 0 \\ \end{array} \right) \\ V= & {} \left( \begin{array}{ccccc} \vartheta &{} \quad \frac{\beta _{h} \varpropto \mu }{\vartheta } &{} \quad 0 &{} \quad \frac{\beta _{e} \varpropto \nu }{\vartheta } &{} \quad 0 \\ 0 &{}\quad \alpha +\vartheta &{} \quad 0 &{} \quad 0 &{}\quad 0 \\ 0 &{}\quad 0 &{}\quad \vartheta +\phi &{}\quad 0 &{} \quad 0 \\ 0 &{} \quad 0 &{} \quad 0 &{} \quad \vartheta &{}\quad 0 \\ 0 &{} \quad 0 &{} \quad 0 &{} \quad 0 &{} \quad \vartheta \\ \end{array} \right) \\ V^{-1}= & {} \left( \begin{array}{ccccc} \frac{\vartheta ^2 (\alpha +\vartheta ) (\vartheta +\phi )}{\alpha \vartheta ^4+\alpha \vartheta ^3 \phi +\vartheta ^5+\vartheta ^4 \phi } &{} \quad \frac{-\beta _{h} \vartheta ^2 \varpropto \mu -\beta _{h} \vartheta \varpropto \mu \phi }{\alpha \vartheta ^4+\alpha \vartheta ^3 \phi +\vartheta ^5+\vartheta ^4 \phi } &{} \quad 0 &{}\quad \frac{-\alpha \beta _{e} \vartheta \varpropto \nu -\alpha \beta _{e} \varpropto \nu \phi -\beta _{e} \vartheta ^2 \varpropto \nu -\beta _{e} \vartheta \varpropto \nu \phi }{\alpha \vartheta ^4+\alpha \vartheta ^3 \phi +\vartheta ^5+\vartheta ^4 \phi } &{}\quad 0 \\ 0 &{}\quad \frac{\vartheta ^4+\vartheta ^3 \phi }{\alpha \vartheta ^4+\alpha \vartheta ^3 \phi +\vartheta ^5+\vartheta ^4 \phi } &{} \quad 0 &{} \quad 0 &{}\quad 0 \\ 0 &{}\quad 0 &{} \quad \frac{\alpha \vartheta ^3+\vartheta ^4}{\alpha \vartheta ^4+\alpha \vartheta ^3 \phi +\vartheta ^5+\vartheta ^4 \phi } &{}\quad 0 &{} 0 \\ 0 &{} \quad 0 &{} \quad 0 &{} \quad \frac{\vartheta \left( \alpha \vartheta +\vartheta ^2\right) (\vartheta +\phi )}{\alpha \vartheta ^4+\alpha \vartheta ^3 \phi +\vartheta ^5+\vartheta ^4 \phi } &{} \quad 0 \\ 0 &{}\quad 0 &{} \quad 0 &{}\quad 0 &{}\quad \frac{\vartheta \left( \alpha \vartheta +\vartheta ^2\right) (\vartheta +\phi )}{\alpha \vartheta ^4+\alpha \vartheta ^3 \phi +\vartheta ^5+\vartheta ^4 \phi } \\ \end{array} \right) \\ K= & {} F.V^{-1} \end{aligned}$$

So,

$$\begin{aligned} K= \left( \begin{array}{ccccc} 0 &{} \quad 0 &{}\quad 0 &{} \quad 0 &{} \quad 0 \\ 0 &{} \frac{\beta _{h} \varpropto \mu \left( \vartheta ^4+\vartheta ^3 \phi \right) }{\vartheta \left( \alpha \vartheta ^4+\alpha \vartheta ^3 \phi +\vartheta ^5+\vartheta ^4 \phi \right) } &{}\quad 0 &{} \quad 0 &{}\quad 0 \\ 0 &{} \frac{\alpha \left( \vartheta ^4+\vartheta ^3 \phi \right) }{\alpha \vartheta ^4+\alpha \vartheta ^3 \phi +\vartheta ^5+\vartheta ^4 \phi } &{} \quad 0 &{}\quad 0 &{} \quad 0 \\ 0 &{}\quad 0 &{}\quad \frac{\sigma \left( \alpha \vartheta ^3+\vartheta ^4\right) }{\alpha \vartheta ^4+\alpha \vartheta ^3 \phi +\vartheta ^5+\vartheta ^4 \phi } &{} \frac{\beta _{e} \varpropto \nu \left( \alpha \vartheta +\vartheta ^2\right) (\vartheta +\phi )}{\alpha \vartheta ^4+\alpha \vartheta ^3 \phi +\vartheta ^5+\vartheta ^4 \phi } &{}\quad 0 \\ 0 &{}\quad 0 &{} \quad \frac{\phi \left( \alpha \vartheta ^3+\vartheta ^4\right) }{\alpha \vartheta ^4+\alpha \vartheta ^3 \phi +\vartheta ^5+\vartheta ^4 \phi } &\quad 0 &\quad 0 \\ \end{array} \right) \end{aligned}$$

Thus

$$\begin{aligned}{} & {} |K - \Upsilon I| = 0\\{} & {} \left| \begin{array}{ccccc} -\Upsilon &{} \quad 0 &{} \quad 0 &{}\quad 0 &{}\quad 0 \\ 0 &{} \frac{\beta _{h} \varpropto \mu \left( \vartheta ^4+\vartheta ^3 \phi \right) }{\vartheta \left( \alpha \vartheta ^4+\alpha \vartheta ^3 \phi +\vartheta ^5+\vartheta ^4 \phi \right) }-\Upsilon &{}\quad 0 &{} \quad 0 &{} \quad 0 \\ 0 &{} \quad \frac{\alpha \left( \vartheta ^4+\vartheta ^3 \phi \right) }{\alpha \vartheta ^4+\alpha \vartheta ^3 \phi +\vartheta ^5+\vartheta ^4 \phi } &{} \quad -\Upsilon &{} \quad 0 &\quad   0 \\ 0 &\quad  0 &\quad  \frac{\sigma \left( \alpha \vartheta ^3+\vartheta ^4\right) }{\alpha \vartheta ^4+\alpha \vartheta ^3 \phi +\vartheta ^5+\vartheta ^4 \phi } &{} \frac{\beta _{e} \varpropto \nu \left( \alpha \vartheta +\vartheta ^2\right) (\vartheta +\phi )}{\alpha \vartheta ^4+\alpha \vartheta ^3 \phi +\vartheta ^5+\vartheta ^4 \phi }-\Upsilon & \quad 0 \\ 0 &{} \quad 0 &{}\quad \frac{\phi \left( \alpha \vartheta ^3+\vartheta ^4\right) }{\alpha \vartheta ^4+\alpha \vartheta ^3 \phi +\vartheta ^5+\vartheta ^4 \phi } &{}\quad 0 &{}\quad -\Upsilon \\ \end{array} \right| =0 \end{aligned}$$

After solving the above determinant matrix, we get the $(\Upsilon )$ as follows

$$\begin{aligned} \Upsilon &=  0, \\ \Upsilon &=  0, \\ \Upsilon &=  0, \\ \Upsilon &=  \frac{\beta _{h} \varpropto \mu }{\vartheta (\alpha +\vartheta )}, \\ \Upsilon &=  \frac{\beta _{e} \varpropto \nu }{\vartheta ^2}. \end{aligned}$$

Since the reproduction number $R_0$ corresponds to the principal eigenvalue of the matrix $FV^{-1}$ as follows:

$$\begin{aligned} R_0=\frac{\beta _{h} \varpropto \mu }{\vartheta (\alpha +\vartheta )}. \end{aligned}$$

### Sensitivity analysis

Sensitivity analysis serves the purpose of assessing the comparative impact of various factors on a models stability, particularly when dealing with uncertain data. Furthermore, this analysis can pinpoint the essential variables in the process. As Reproductive No. is

$$\begin{aligned} R_0 = \frac{\beta _{h} \varpropto \mu }{\vartheta (\alpha +\vartheta )}. \end{aligned}$$

We can analyze the sensitivity of $R_0$ by calculating the partial derivatives of the threshold concerning the relevant parameters as follows:

$$\begin{aligned} \frac{\partial R_0}{\partial \beta _{h}}= & {} \frac{\varpropto \mu }{\vartheta (\alpha +\vartheta )}> 0,\\ \frac{\partial R_0}{\partial \varpropto }= & {} \frac{\beta _{h} \mu }{\vartheta (\alpha +\vartheta )}> 0,\\ \frac{\partial R_0}{\partial \mu }= & {} \frac{\beta _{h} \varpropto }{\vartheta (\alpha +\vartheta )} > 0,\\ \frac{\partial R_0}{\partial \vartheta }= & {} -\frac{\beta _{h} \varpropto \mu }{\vartheta ^2 (\alpha +\vartheta )}-\frac{\beta _{h} \varpropto \mu }{\vartheta (\alpha +\vartheta )^2}< 0,\\ \frac{\partial R_0}{\partial \alpha }= & {} -\frac{\beta _{h} \varpropto \mu }{\vartheta (\alpha +\vartheta )^2} < 0. \end{aligned}$$

As we vary the parameters, we notice that the value of $R_{0}$ is highly responsive. In our study, the parameters $n,\varpropto , \mu$ and $\beta _{h}$ exhibit expansion, while $\vartheta , \alpha$ experience contraction. Consequently, prioritizing prevention over treatment is recommended for effective infection control. We also notice that the value of $R_{0}$ is highly responsive as can be seen in all above sub-figures that how its rate of change behaves. But we can observe from all sub-figures that the rate of change of each parameter comes bounded which is important for stable situation.

## Flip bifurcation analysis

By bifurcation theory^61–63^, we shall give whole bifurcation investigate at $\digamma _1(\frac{\varpropto }{\vartheta }, 0, 0, 0, 0)$ and $\digamma ^+_{SEIBR}$ here by bifurcation.

*Bifurcation analysis about* $\digamma _1(\frac{\varpropto }{\vartheta }, 0, 0, 0, 0)$

From eigen values, the calculation yields $\lambda _\kappa \ne -1,1, \kappa =1,2,3,4$ which indicates that model may go through flip bifurcation if $(\varpropto ,\nu ,\vartheta , \mu , \phi , \alpha , \beta _{h}, \beta _{e} )$ located in the set:

20 $$\begin{aligned} F|_{\digamma _1\left( \frac{\varpropto }{\vartheta ^q}, 0, 0, 0, 0\right) }=\left\{ {\varpropto ,\nu ,\vartheta , \mu , \phi , \alpha ,\beta _{h}, \beta _{e} }:v^q=-\frac{\vartheta ^q }{2}\right\} \end{aligned}$$

Butt following theorem states that if $(\varpropto ,\nu ,\vartheta , \mu , \phi , \alpha , \beta _{h}, \beta _{e} ) \in F|_{\digamma _1}(\frac{\varpropto }{ \vartheta }, 0, 0, 0, 0)$, So there is no flip bifurcation occur for model at $F|_{\digamma _1}(\frac{\varpropto }{ \vartheta }, 0, 0, 0, 0)$.

### Theorem 4

If $(\varpropto ,\nu ,\vartheta , \mu , \phi , \alpha , \beta _{h}, \beta _{e} ) \in F|_{\digamma _{1}}(\frac{\varpropto }{ \vartheta }, 0, 0, 0, 0)$ then no flip bifurcation exists for model at $F|_{\digamma _{1}}(\frac{\varpropto }{\vartheta }, 0, 0, 0, 0)$.

### Proof

The model is steady and therefore its order is forbidden to analyze is known as bifuracation with the condition $E=B=I=R=0$ which gives this shape

21 $$\begin{aligned} S_{t+1}= \varpropto -(\vartheta )S \end{aligned}$$

From (22), one denotes the map

22 $$\begin{aligned} f(S)= \varpropto - (\vartheta )S \end{aligned}$$

Now if $\vartheta =\vartheta ^*=-\frac{\phi }{2}$ and $S=S^*=\frac{\varpropto }{\vartheta }$ From (22), one denotes the map

$$\begin{aligned} \frac{\partial f}{\partial S}\Bigg |_{\vartheta =\vartheta ^*=-\frac{\phi }{2},S = S^*= \frac{\varpropto }{\vartheta }}= & {} -(\vartheta ) = -\left( -\frac{\phi }{2}\right) =\frac{\phi }{2}\ne 0 \\ \frac{\partial f}{\partial \vartheta }\Bigg |_{\vartheta ^q=\vartheta ^*=-\frac{\phi }{2},S = S^*= \frac{\varpropto }{\vartheta }}= & {} -(S) =-\frac{\varpropto }{\vartheta }=-\frac{2\varpropto }{\phi } \end{aligned}$$

and

23 $$\begin{aligned} \frac{\partial f^2}{\partial ^2 S}\Bigg |_{\vartheta =\vartheta ^*=-\frac{\phi }{2},S=S^*=\frac{\varpropto }{\vartheta }} = 0. \end{aligned}$$

The portion shows that at $\digamma _{1}(\frac{\varpropto }{\vartheta ^q}, 0, 0, 0, 0)$ of model there exists no flip bifurcation because counted parametric conditions (23) oppose the situation of non-degenerate for presence of flip bifurcation if $(\varpropto ,\nu ,\vartheta , \mu , \phi , \alpha , \beta _{h}, \beta _{e}) \in F|_{\digamma _{1}}(\frac{\varpropto }{ \vartheta }, 0, 0, 0, 0)$. $\square$

**Figure 2:**
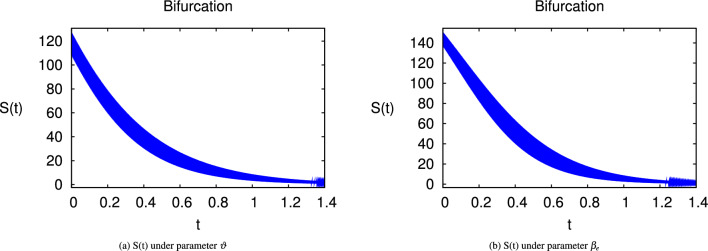
Bifurcation analysis of continuous dynamics for *S*(*t*).

**Figure 3:**
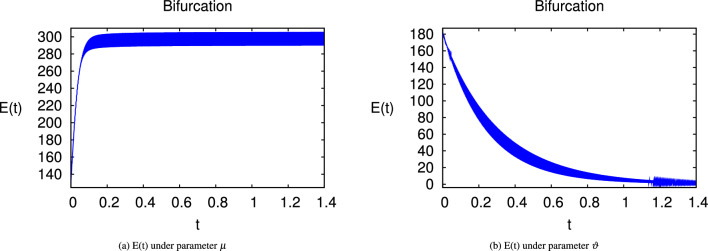
Bifurcation analysis of continuous dynamics for *E*(*t*).

**Figure 4:**
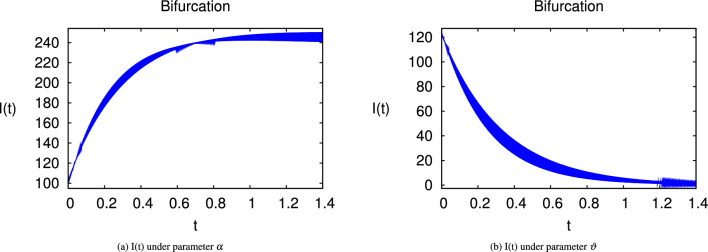
Bifurcation analysis of continuous dynamics for *I*(*t*).

**Figure 5:**
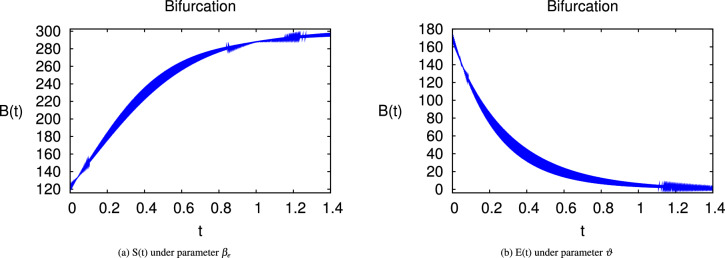
Bifurcation analysis of continuous dynamics for *B*(*t*).

**Figure 6:**
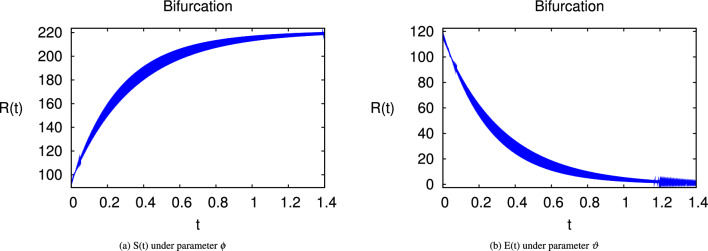
Bifurcation analysis of continuous dynamics for *R*(*t*).

Here we have newly developed cholera disease model which is investigated with the help of asymptomatic measures. It has immense complex consequences on population here, we have a continuous time system for cholera interaction. It has hypotheses that parameters used in this model are $\varpropto = 0.00005480, \mu =0.01, \beta _h =0.04444, \beta _e =0.124, \alpha = 0.034,\sigma = 0.0006, \phi = 0.029$ and $\nu = 0.02$. Here we have constructed the bifurcation diagram of continuous time graph of model with respect of different parametric values in specific ranges which gives the stable state of cholera disease model by introducing the asymptomatic measures. Our theocratical results are supported by Fig. 2, 3, 4, 5, 6, with the help of time steady graphs in which rate of parametric values of total requitement rate.

## Solution by advanced numerical scheme

We will develop the numerical scheme in this section while applying our recommended method to approximate the fractional partial differential equation. In the next theorem, this will be proven. The following statements true for the numerical scheme for the model of cholera disease (7).

Fundamental theorem of the fractional calculus may be employing to transform the equation of $S = \varpropto - \beta _{h} \mu E(t) S(t) - \beta _{e} \nu B(t) S(t) - \vartheta S(t)$ of (7) into the fractional integral equation:

24 $$\begin{aligned} S(t)-S(0)= & {} \frac{1-\xi }{ABC(\xi )} \Big (\varpropto - \mu \beta _{h} S(t) E(t) - \nu \beta _{e} S(t) B(t) - \vartheta S(t) \Big ) + \frac{\xi }{\Gamma (\xi ) \times ABC(\xi )} \nonumber \\{} & {} \times \int _{0}^{t} \Big (\varpropto - \mu \beta _{h} S(\zeta ) E(\zeta ) - \nu \beta _{e} S(\zeta ) B(\zeta ) - \vartheta S(\zeta ) \Big ) (t-\zeta )^{\xi -1} d\zeta . \end{aligned}$$

The aforementioned equation is rewritten as follows at a particular point $=t_{k+1}, k = 0,1,2,...,$:

25 $$\begin{aligned} S(t_{k+1})-S(0)= & {} \frac{1-\xi }{ABC(\xi )} \Big (\varpropto - \mu \beta _{h} S(t_k) E(t_k) - \nu \beta _{e} S(t_k) B(t_k) - \vartheta S(t_k) \Big ) + \frac{\xi }{\Gamma (\xi ) \times ABC(\xi )} \nonumber \\{} & {} \times \int _{0}^{t_{k+1}} \Big (\varpropto - \mu \beta _{h} S(\zeta ) E(\zeta ) - \nu \beta _{e} S(\zeta ) B(\zeta ) - \vartheta S(\zeta ) \Big ) (t_{k+1}-\zeta )^{\xi -1} d\zeta \nonumber \\= & {} \frac{1-\xi }{ABC(\xi )} \Big (\varpropto - \mu \beta _{h} S(t_k) E(t_k) - \nu \beta _{e} S(t_k) B(t_k) - \vartheta S(t_k) \Big ) + \frac{\xi }{ABC(\xi ) \times \Gamma (\xi )} \nonumber \\{} & {} \times \sum _{\chi =0}^{k} \int _{t_\chi }^{t_{\chi +1}} \Big (\varpropto - \mu \beta _{h} S(\zeta ) E(\zeta ) - \nu \beta _{e} S(\zeta ) B(\zeta ) - \vartheta S(\zeta ) \Big ) (t_{k+1}-\zeta )^{\xi -1} d\zeta . \end{aligned}$$

Within the interval $[t_\chi , t_{\chi +1}]$, the function $\varpropto - \mu \beta _{h} S(\zeta ) E(\zeta ) - \nu \beta _{e} S(\zeta ) B(\zeta ) - \vartheta S(\zeta )$, Employing the two step lagrange polynomial interpolation,may be estimated like this:

$$\begin{aligned} P_\chi (\zeta )= & {} \frac{\zeta - t_{\chi -1}}{t_\chi - t_{\chi -1}} \Big (\varpropto - \mu \beta _{h} S(t_\chi ) E(t_\chi ) - \nu \beta _{e} S(t_\chi ) B(t_\chi ) - \vartheta S(t_\chi ) \Big ) \\{} & {} -\frac{\zeta - t_{\chi }}{t_\chi - t_{\chi -1}} \Big (\varpropto - \mu \beta _{h} S(t_\chi -1) E(t_\chi -1) - \nu \beta _{e} S(t_\chi -1) B(t_\chi -1) - \vartheta S(t_\chi -1) \Big )\ \\= & {} \frac{\varpropto - \mu \beta _{h} S(t_\chi ) E(t_\chi ) - \nu \beta _{e} S(t_\chi ) B(t_\chi ) - \vartheta S(t_\chi ) }{h} (\zeta -t_{\chi -1}) \\{} & {} -\frac{\varpropto - \mu \beta _{h} S(t_\chi -1) E(t_\chi -1) - \nu \beta _{e} S(t_\chi -1) B(t_\chi -1) - \vartheta S(t_\chi -1) }{h} (\zeta - t_{\chi }) \ \\\simeq & {} \frac{\varpropto - \mu \beta _{h} S(t_\chi ) E(t_\chi ) - \nu \beta _{e} S(t_\chi ) B(t_\chi ) - \vartheta S(t_\chi ) }{h} (\zeta -t_{\chi -1}) \ \\{} & {} - \frac{\varpropto - \mu \beta _{h} S(t_\chi -1) E(t_\chi -1) - \nu \beta _{e} S(t_\chi -1) B(t_\chi -1) - \vartheta S(t_\chi -1) }{h} (\zeta - t_{\chi }). \ \end{aligned}$$

Thus, the aforementioned estimate may be included in Eq. (25) in order to generate.

26 $$\begin{aligned} (S)_{k+1}= & {} (S)_0 + \frac{1-\xi }{ABC(\xi )} \Big (\varpropto - \mu \beta _{h} S(t_k) E(t_k) - \nu \beta _{e} S(t_k) B(t_k) - \vartheta S(t_k) \Big ) + \frac{\xi }{ABC(\xi ) \times \Gamma (\xi )} \nonumber \\{} & {} \times \sum _{\chi =0}^{k}\Bigg (\frac{\big ( \varpropto - \mu \beta _{h} S(\chi ) E(\chi ) - \nu \beta _{e} S(\chi ) B(\chi ) - \vartheta S(\chi ) \big )}{h} \int _{t_\chi }^{t_{\chi +1}}(\zeta -t_{\chi -1}) (t_{k+1}-\zeta )^{\xi -1} d\zeta - \end{aligned}$$

27 $$ \frac{{ \propto  - \mu \beta _{h} S(\chi  - 1)E(\chi  - 1) - \nu \beta _{e} S(\chi  - 1)B(\chi  - 1) - \vartheta S(\chi  - 1)}}{h}\int_{{t_{\chi } }}^{{t_{{\chi  + 1}} }} {(\zeta  - t_{\chi } )(t_{{k + 1}}  - \zeta )^{{\xi  - 1}} } d\zeta ).{\text{ }} $$

For simplicity, we let

28 $$\begin{aligned} A_{\xi ,1,\chi } = \int _{t_\chi }^{t_{1+\chi }}(\zeta -t_{\chi -1}) (t_{k+1}-\zeta )^{\xi -1} d\zeta \end{aligned}$$

and also

29 $$\begin{aligned} A_{\xi ,2,\chi }= & {} \int _{t_\chi }^{t_{1+\chi }}(\zeta -t_{\chi }) (t_{k+1}-\zeta )^{\xi -1} d\zeta \nonumber \\ A_{\xi ,1,\chi }= & {} h^{\xi +1} \frac{(k+\chi +1)^\xi (k+2+\xi -\chi ) - (k-\chi )^\xi (k+2+2\xi -\chi )}{\xi (\xi +1)}\nonumber \\ A_{\xi ,\chi ,2}= & {} h^{\xi +1} \frac{(k+\chi +1)^{\xi +1} - (k-\chi )^\xi (k+1+\xi -\chi )}{\xi (\xi +1)}. \end{aligned}$$

By integrating Eqs. (28) and (29) and substituting them with Eq. (26), we can gain:

$$\begin{aligned} (S)_{k+1}= & {} (S)_0 + \frac{1-\xi }{ABC(\xi )} \Big (\varpropto - \mu \beta _{h} S(t_k) E(t_k) - \nu \beta _{e} S(t_k) B(t_k) - \vartheta S(t_k) \Big ) + \frac{\xi }{ABC(\xi ) \times \Gamma (\xi )} \ \\{} & {} \times \sum _{\chi =0}^{k}\bigg (\frac{h^\xi \big ( \varpropto - \mu \beta _{h} S(\chi ) E(\chi ) - \nu \beta _{e} S(\chi ) B(\chi ) - \vartheta S(\chi ) \big )}{\Gamma (\xi +2)} (k-\chi +1)^\xi (k+2+\xi -\chi )\\{} & {} -\frac{h^\xi (\varpropto - \mu \beta _{h} E(\chi -1) S(\chi -1) - \nu \beta _{e} B(\chi -1) S(\chi -1) - \vartheta S(\chi -1) )}{\Gamma (\xi +2)} \big ((k-\chi +1)^{\xi +1} \\{} & {} -(k-\chi )^\xi (k+1+\xi -\chi )\big ) \bigg ). \end{aligned}$$

Fundamental theorem of the fractional calculus may be employing to transform the equation of $E =\mu \beta _{h} S(t) E(t) - \alpha E(t) - \vartheta E(t)$ of (7) into the fractional integral equation:

30 $$\begin{aligned} E(t)-E(0)= & {} \frac{1-\xi }{ABC(\xi )} \Big (\mu \beta _{h} S(t) E(t) - \alpha E(t) - \vartheta E(t)\Big ) \nonumber \\{} & {} + \frac{\xi }{\Gamma (\xi ) \times ABC(\xi )}\int _{0}^{t} \Big (\mu \beta _{h} S(\zeta ) E(\zeta ) - \alpha E(\zeta ) - \vartheta E(\zeta )\Big ) (t-\zeta )^{\xi -1} d\zeta . \end{aligned}$$

The aforementioned equation is rewritten as follows at a particular point $=t_{k+1}, k = 0,1,2,\ldots ,$:

31 $$\begin{aligned} E(t_{k+1})-E(0)= & {} \frac{1-\xi }{ABC(\xi )}\Big (\mu \beta _{h}S(t_k) E(t_k) -\alpha E(t_k) - \vartheta E(t_k)\Big ) \nonumber \\{} & {} \times \frac{\xi }{\Gamma (\xi ) \times ABC(\xi )} \int _{0}^{t_{k+1}} +\Big (\mu \beta _{h} S(\zeta ) E(\zeta ) - \alpha E(\zeta ) - \vartheta E(\zeta )\Big ) (t_{k+1}-\zeta )^{\xi -1} d\zeta \nonumber \\= & {} \frac{1-\xi }{ABC(\xi )} \Big (\mu \beta _{h} S(t_k) E(t_k) - \alpha E(t_k) - \vartheta E(t_k)\Big )\nonumber \\{} & {} +\frac{\xi }{ABC(\xi ) \times \Gamma (\xi )} \sum _{\chi =0}^{k} \int _{t_\chi }^{t_{\chi +1}} \Big (\mu \beta _{h} S(\zeta ) E(\zeta ) - \alpha E(\zeta ) - \vartheta E(\zeta )\Big ) (t_{k+1}-\zeta )^{\xi -1} d\zeta . \end{aligned}$$

Within the interval $[t_\chi , t_{\chi +1}]$, the function $\Big (\mu \beta _{h} S(\zeta ) E(\zeta ) - \alpha E(\zeta ) - E(\zeta )\Big )$, Employing the two step lagrange polynomial interpolation,may be estimated like this:

$$\begin{aligned} P_\chi (\zeta )= & {} \frac{\zeta - t_{\chi -1}}{t_\chi - t_{\chi -1}} \Big (\mu \beta _{h} S(t_\chi ) E(t_\chi ) - \alpha E(t_\chi ) - \vartheta E(t_\chi )\Big ) \\{} & {} - \frac{\zeta - t_{\chi }}{t_\chi - t_{\chi -1}}\Big (\mu \beta _{h} S(t_{\chi -1}) E(t_{\chi -1}) - \alpha E(t_{\chi -1}) - \vartheta E(t_{\chi -1})\Big )\ \\= & {} \frac{\mu \beta _{h} S(t_\chi ) E(t_\chi ) - \alpha E(t_\chi ) - \vartheta E(t_\chi )}{h} (\zeta -t_{\chi -1}) \\{} & {} - \frac{\mu \beta _{h} S(t_{\chi -1}) E(t_{\chi -1}) - \alpha E(t_{\chi -1}) - \vartheta E(t_{\chi -1})}{h} (\zeta - t_{\chi }) \ \\\simeq & {} \frac{\Big (\mu \beta _{h} S(\chi ) E(\chi ) - \alpha E(\chi ) - \vartheta E(\chi )\Big )}{h} (\zeta -t_{\chi -1})\\{} & {} - \frac{\Big (\mu \beta _{h} S({\chi -1}) E({\chi -1}) - \alpha E({\chi -1}) - \vartheta E({\chi -1})\Big )}{h} (\zeta - t_{\chi }). \ \end{aligned}$$

Thus, the aforementioned estimate may be included in Eq. (31) in order to generate.

32 $$\begin{aligned} (E)_{k+1}= & {} (E)_0 + \frac{(1-\xi )}{ABC(\xi )} \Big (\mu \beta _{h} S(t_k) E(t_k) - 
\alpha E(t_k) - \vartheta E(t_k)\Big ) \nonumber \\{} & {} + \frac{\xi }{ABC(\xi ) \times \Gamma (\xi )} \sum _{\chi =0}^{k} \Big ( \frac{\big (\mu \beta _{h} S(\chi ) E(\chi ) - \alpha E(\chi ) - \vartheta E(\chi )\big )}{h} \nonumber \\{} & {} \times \int _{t_\chi }^{t_{\chi +1}} (\zeta - t_{\chi -1})(t_{k+1} - \zeta )^{\xi -1}d\zeta - \frac{\big (\mu \beta _{h} S(\chi -1) E(\chi -1) - \alpha E(\chi -1) - \vartheta E(\chi -1)\big )}{h}\nonumber \\{} & {} \times \int _{t_\chi }^{t_{\chi +1}} (\zeta - t_{\chi })(t_{k+1} - \zeta )^{\xi -1}d\zeta \Big ). \end{aligned}$$

For simplicity, we let

33 $$\begin{aligned} A_{\xi ,\chi ,1} = \int _{t_\chi }^{t_{\chi +1}} (\zeta - t_{\chi -1})(t_{k+1} - \zeta )^{\xi -1}d\zeta \end{aligned}$$

and also

34 $$\begin{aligned} A_{\xi ,\chi ,2}= & {} \int _{t_\chi }^{t_{\chi +1}} (\zeta - t_{\chi })(t_{n+1} - \zeta )^{\xi -1}d\zeta \nonumber \\ A_{\xi ,\chi ,1}= & {} \frac{\left( h^{\xi +1}\right) \left\{ (k+1+\chi )^\xi (k-\chi +2+\xi ) - (k-\chi )^\xi (k-\chi +2+2\xi )\right\} }{\xi (\xi +1)}\nonumber \\ A_{\xi ,\chi ,2}= & {} \frac{ \left( h^{\xi +1}\right) \left\{ (k+1+\chi )^{\xi +1} - (k-\chi )^\xi (k-\chi +1+\xi )\right\} }{\xi (\xi +1)}. \end{aligned}$$

By integrating Eqs. (33) and (34) and substituting them with Eq. (32), we get

$$\begin{aligned} (E)_{k+1}= & {} (E)_0 + \frac{1-\xi }{ABC(\xi )} \Big (\mu \beta _{h} S(t_k) E(t_k) - \alpha E(t_k) - \vartheta E(t_k)\Big ) + \frac{\xi }{ABC(\xi )} \\{} & {} \times \sum _{\chi =0}^{k} \Bigg (\frac{h^\xi \Big (\mu \beta _{h} S(\chi ) E(\chi ) - \alpha E(\chi ) - \vartheta E(\chi )\Big )}{\Gamma (\xi +2)} ((k+1-\chi )^\xi (k-\chi +2+\xi )) \\{} & {} -\frac{h^\xi \Big (\mu \beta _{h} S(\chi -1) E(\chi -1) - \alpha E(\chi -1) - \vartheta E(\chi -1)\Big )}{\Gamma (\xi +2)} \\{} & {} \times \Big ((k+1-\chi )^{\xi +1} -(k-\chi )^\xi (k-\chi +1+\xi )\Big ) \Bigg ). \end{aligned}$$

Fundamental theorem of the fractional calculus may be employing to transform the equation of $I=\alpha E(t) - \phi I(t) - \vartheta I(t)$ of (7)into the fractional integral equation:

$$\begin{aligned} I(t)-I(0)= & {} \frac{1-\xi }{ABC(\xi )}\Big (\alpha E(t) - \phi I(t) - \vartheta I(t)\Big )\\{} & {} + \frac{\xi }{\Gamma (\xi ) \times ABC(\xi )} \int _{0}^{t} \Big (\alpha E(\zeta ) - \phi I(\zeta ) - \vartheta I(\zeta )\Big ) (t-\zeta )^{\xi -1} d\zeta . \end{aligned}$$

The aforementioned equation is rewritten as follows at a particular point $=t_{k+1}, k = 0,1,2,\ldots ,$:

35 $$\begin{aligned} I(t_{k+1})-I(0)= & {} \frac{1-\xi }{ABC(\xi )} \Big (\alpha E(t_k) - \phi I(t_k) - \vartheta I(t_k)\Big ) \nonumber \\{} & {} + \frac{\xi }{\Gamma (\xi ) \times ABC(\xi )} \int _{0}^{t_{k+1}} \Big (\alpha E(\zeta ) - \phi I(\zeta ) - \vartheta I(\zeta )\Big )(t_{n+1}-\zeta )^{\xi -1} d\zeta \nonumber \\= & {} \frac{1-\xi }{ABC(\xi )} \Big (\alpha E(t_k) - \phi I(t_k) - \vartheta I(t_k)\Big ) \nonumber \\{} & {} + \frac{\xi }{ABC(\xi ) \times \Gamma (\xi )} \sum _{\chi =0}^{k} \int _{t_\chi }^{t_{\chi +1}} \Big (\alpha E(\zeta ) - \phi I(\zeta ) - \vartheta I(\zeta )\Big ) (t_{k+1}-\zeta )^{\xi -1} d\zeta . \end{aligned}$$

Within the interval $[t_\chi , t_{\chi +1}]$, the function $\Big (\alpha E(\zeta ) - \phi I(\zeta ) - \vartheta I(\zeta )\Big )$, Employing the two step lagrange polynomial interpolation, may be estimated like this:

36 $$\begin{aligned} P_\chi (\zeta )= & {} \frac{\zeta - t_{\chi -1}}{t_\chi - t_{\chi -1}} \Big (\alpha E(t_\chi ) - \phi I(t_\chi ) - \vartheta I(t_\chi )\Big ) - \frac{\zeta - t_{\chi }}{t_\chi - t_{\chi -1}} \Big (\alpha E(t_{\chi -1}) - \phi I(t_{\chi -1}) - \vartheta I(t_{\chi -1})\Big ) \nonumber \\= & {} \frac{\Big (\alpha E(t_\chi ) - \phi I(t_\chi ) - \vartheta I(t_\chi )\Big )}{h} (\zeta -t_{\chi -1}) - \frac{\Big (\alpha E(t_{\chi -1}) - \phi I(t_{\chi -1}) - \vartheta I(t_{\chi -1})\Big )}{h} (\zeta - t_{\chi }) \nonumber \\\simeq & {} \frac{\Big (\alpha E(\chi ) - \phi I(\chi ) - \vartheta I(\chi )\Big )}{h} (\zeta -t_{k-1}) - \frac{\Big (\alpha E(\chi -1) - \phi I(\chi -1) - \vartheta I(\chi -1)\Big )}{h} (\zeta - t_{\chi }). \end{aligned}$$

Thus, the aforementioned estimate may be included in Eq. (35) in order to generate.

37 $$\begin{aligned} (I)_{k+1}= & {} (I)_0 + \frac{(1-\xi )}{ABC(\xi )} \Big (\alpha E(t_k) - \phi I(t_k) - \vartheta I(t_k)\Big ) \nonumber \\{} & {} + \frac{\xi }{ABC(\xi ) \times \Gamma (\xi )} \sum _{\chi =0}^{k} \Bigg ( \frac{\big (\alpha E(\chi ) - \phi I(\chi ) - \vartheta I(\chi )\big )}{h} \nonumber \\{} & {} \times \int _{t_\chi }^{t_{\chi +1}} (\zeta - t_{\chi -1})(t_{k+1} - \zeta )^{\xi -1}d\zeta \nonumber \\{} & {} - \frac{\big (\alpha E(\chi -1) - \phi I(\chi -1) - \vartheta I(\chi -1)\big )}{h} \int _{t_\chi }^{t_{\chi +1}} (\zeta - t_{\chi })(t_{k+1} - \zeta )^{\xi -1}d\zeta \Bigg ). \end{aligned}$$

For simplicity, we let

38 $$\begin{aligned} A_{\xi ,\chi ,1} = \int _{t_\chi }^{t_{\chi +1}} (\zeta - t_{\chi -1})(t_{k+1} - \zeta )^{\xi -1}d\zeta \end{aligned}$$

and also

39 $$\begin{aligned} A_{\xi ,\chi ,2}= & {} \int _{t_\chi }^{t_{\chi +1}} (\zeta - t_{\chi })(t_{k+1} - \zeta )^{\xi -1}d\zeta \nonumber \\ A_{\xi ,\chi ,1}= & {} \frac{(h^{\xi +1})\{(n+1+\chi )^\xi (k-\chi +2+\xi ) - (k-\chi )^\xi (k-\chi +2+2\xi )\}}{\xi (\xi +1)}\nonumber \\ A_{\xi ,\chi ,2}= & {} \frac{(h^{\xi +1})\{(k+1+\chi )^{\xi +1} - (k-\chi )^\xi (k-\chi +1+\xi )\}}{\xi (\xi +1)}. \end{aligned}$$

By integrating Eqs. (38) and (39) and substituting them with Eq. (37), we can gain:

$$\begin{aligned} (I)_{k+1}= & {} (I)_0 + \frac{1-\xi }{ABC(\xi )} \Big (\alpha E(t_k) - \phi I(t_k) - \vartheta I(t_k)\Big ) \\{} & {} + \frac{\xi }{ABC(\xi )} \sum _{\chi =0}^{k} \Bigg (\frac{h^\xi \Big (\alpha E(\chi ) - \phi I(\chi ) - \vartheta I(\chi )\Big )}{\Gamma (\xi +2)}(k+1-\chi )^\xi (k-\chi +2+\xi ) \\{} & {} -\frac{h^\xi \Big (\alpha E(\chi -1) - \phi I(\chi -1) - \vartheta I(\chi -1)\Big )}{\Gamma (\xi +2)}\big ((k+1-\chi )^{\xi +1} \\{} & {} - (k-\chi )^\xi (k-\chi +1+\xi )\big )\Bigg ). \end{aligned}$$

Fundamental theorem of the fractional calculus may be employing to transform the equation of $B=\sigma I(t) + \nu \beta _{e} S(t) B(t) - \vartheta B(t)$ of (7) into the fractional integral equation:

40 $$\begin{aligned} B(t)-B(0)= & {} \frac{1-\xi }{ABC(\xi )} \Big (\sigma I(t) + \nu \beta _{e} S(t) B(t) - \vartheta B(t)\Big ) + \frac{\xi }{\Gamma (\xi ) \times ABC(\xi )} \nonumber \\{} & {} \times \int _{0}^{t} \Big (\sigma I(\zeta ) + \nu \beta _{e} S(\zeta ) B(\zeta ) - \vartheta B(\zeta )\Big ) (t-\zeta )^{\xi -1} d\zeta . \end{aligned}$$

The aforementioned equation is rewritten as follows at a particular point $=t_{k+1}, k = 0,1,2,\ldots ,$:

41 $$\begin{aligned} B(t_{k+1}) - B(0)= & {} \frac{1-\xi }{ABC(\xi )} \Big (\sigma I(t_k) + \nu \beta _{e} S(t_k) B(t_k) - \vartheta B(t_k)\Big ) + \frac{\xi }{\Gamma (\xi ) \times ABC(\xi )} \nonumber \\{} & {} \times \int _{0}^{t_{k+1}} \Big (\sigma I(\zeta ) + \nu \beta _{e} S(\zeta ) B(\zeta ) - \vartheta B(\zeta )\Big ) (t_{k+1}-\zeta )^{\xi -1} d\zeta \nonumber \\= & {} \frac{1-\xi }{ABC(\xi )} \Big (\sigma I(t_k) + \nu \beta _{e} S(t_k) B(t_k) - \vartheta B(t_k)\Big ) + \frac{\xi }{ABC(\xi ) \times \Gamma (\xi )} \nonumber \\{} & {} \times \sum _{\chi =0}^{k} \int _{t_\chi }^{t_{\chi +1}} \Big (\sigma I(\zeta ) + \nu \beta _{e} S(\zeta ) B(\zeta ) - \vartheta (t_{k+1}-\zeta )^{\xi -1} d\zeta . \end{aligned}$$

Within the interval $[t_\chi , t_{\chi +1}]$, the function $\Big (\sigma I(\zeta ) + \nu \beta _{e} S(\zeta ) B(\zeta ) - B(\zeta )\Big )$, Employing the two step lagrange polynomial interpolation,may be estimated like this:

42 $$\begin{aligned} P_\chi (\zeta )= & {} \frac{\zeta - t_{\chi -1}}{t_\chi - t_{\chi -1}} \Big (\sigma I(t_\chi ) + \nu \beta _{e} S(t_\chi ) B(t_\chi ) - \vartheta B(t_\chi )\Big ) \nonumber \\{} & {} - \frac{\zeta - t_{\chi }}{t_\chi - t_{\chi -1}} \Big (\sigma I(t_{\chi -1}) + \nu \beta _{e} S(t_{\chi -1}) B(t_{\chi -1}) - \vartheta \vartheta B(t_{\chi -1})\Big )\nonumber \\= & {} \frac{\Big (\sigma I(t_\chi ) + \nu \beta _{e} S(t_\chi ) B(t_\chi ) - \vartheta B(t_\chi )\Big )}{h}(\zeta - t_{\chi -1})\nonumber \\{} & {} - \frac{\Big (\sigma I(t_{\chi -1}) + \nu \beta _{e} S(t_{\chi -1}) B(t_{\chi -1}) - \vartheta B(t_{\chi -1})\Big )}{h} (\zeta - t_{\chi }) \nonumber \\\simeq & {} \frac{\Big (\sigma I(\chi ) + \nu \beta _{e} S(\chi ) B(\chi ) - \vartheta B(\chi )\Big )}{h} (\zeta -t_{\chi -1}) \nonumber \\{} & {} -\frac{\Big (\sigma I(\chi -1) + \nu \beta _{e} S(\chi -1) B(\chi -1) - \vartheta B(\chi -1)\Big )}{h} (\zeta - t_{\chi }). \end{aligned}$$

Thus, the aforementioned estimate may be included in Eq. (41) in order to generate.

43 $$\begin{aligned} (B)_{k+1}= & {} (B)_0 + \frac{(1-\xi )}{ABC(\xi )} \Big (\sigma I(t_k) + \nu \beta _{e} S(t_k) B(t_k) - \vartheta B(t_k)\Big ) + \frac{\xi }{ABC(\xi ) \times \Gamma (\xi )} \nonumber \\{} & {} \times \sum _{\chi =0}^{k} \Bigg ( \frac{\big (\sigma I(\chi ) + \nu \beta _{e} S(\chi ) B(\chi ) - \vartheta B(\chi )\big )}{h} \int _{t_\chi }^{t_{\chi +1}} (\zeta - t_{\chi -1})(t_{k+1} - \zeta )^{\xi -1}d\zeta \nonumber \\{} & {} -\frac{\Big (\sigma I(\chi -1) + \nu \beta _{e} S(\chi -1) B(\chi -1) - \vartheta B(\chi -1)\Big )}{h} \int _{t_\chi }^{t_{\chi +1}} (\zeta - t_{\chi })(t_{k+1} - \zeta )^{\xi -1}d\zeta \Bigg ). \end{aligned}$$

For simplicity, we let

44 $$\begin{aligned} A_{\xi ,\chi ,1} = \int _{t_\chi }^{t_{\chi +1}} (\zeta - t_{\chi -1})(t_{k+1} - \zeta )^{\xi -1}d\zeta \end{aligned}$$

and also

45 $$\begin{aligned} A_{\xi ,\chi ,2}= & {} \int _{t_\chi }^{t_{\chi +1}} (\zeta - t_{\chi })(t_{k+1} - \zeta )^{\xi -1}d\zeta \nonumber \\ A_{\xi ,\chi ,1}= & {} \frac{(h^{\xi +1})\{(k+1+\chi )^\xi (k-\chi +2+\xi ) - (k-\chi )^\xi (k-\chi +2+2\xi )\}}{\xi (\xi +1)}\nonumber \\ A_{\xi ,\chi ,2}= & {} \frac{(h^{\xi +1})\{(k+1+\chi )^{\xi +1} - (k-\chi )^\xi (k-\chi +1+\xi )\}}{\xi (\xi +1)}. \end{aligned}$$

By integrating Eqs. (44) and (45) and substituting them with Eq. (43), we can gain:

$$\begin{aligned} (B)_{k+1}= & {} (B)_0 + \frac{1-\xi }{ABC(\xi )} \Big (\sigma I(t_k) + \nu \beta _{e} S(t_k) B(t_k) - \vartheta B(t_k)\Big ) \\{} & {} + \frac{\xi }{ABC(\xi )} \sum _{\chi =0}^{k} \Bigg (\frac{h^\xi \Big (\sigma I(\chi ) + \nu \beta _{e} S(\chi ) B(\chi ) - \vartheta B(\chi )\Big )}{\Gamma (\xi +2)} ((k+1-\chi )^\xi (k-\chi +2+\xi ))\\{} & {} -\frac{h^\xi \Big (\sigma I(\chi -1)+\nu \beta _{e} S(\chi -1) B(\chi -1) - \vartheta B(\chi -1)\Big )}{\Gamma (\xi +2)}\Big ((k+1-\chi )^{\xi +1} \\{} & {} -(k-\chi )^\xi (k-\chi +1+\xi )\Big ) \Bigg ). \end{aligned}$$

Fundamental theorem of the fractional calculus may be employing to transform the equation of $R = \phi I(t) - \vartheta R(t)$ of (7) into the fractional integral equation:

46 $$\begin{aligned} R(t) - R(0)= & {} \frac{1-\xi }{ABC(\xi )} \Big (\phi I(t) - \vartheta R(t)\Big ) + \frac{\xi }{\Gamma (\xi ) \times ABC(\xi )} \int _{0}^{t} \Big (\phi I(\zeta ) - \vartheta R(\zeta )\Big ) (t-\zeta )^{\xi -1} d\zeta . \end{aligned}$$

The aforementioned equation is rewritten as follows at a particular point $=t_{k+1}, k = 0,1,2,\ldots ,$:

47 $$\begin{aligned} R(t_{k+1})-R(0)= & {} \frac{1-\xi }{ABC(\xi )} \Big (\phi I(t_k) - \vartheta R(t_k)\Big ) \nonumber \\{} & {} + \frac{\xi }{\Gamma (\xi ) \times ABC(\xi )} \int _{0}^{t_{k+1}} \Big (\phi I(\zeta ) - \vartheta R(\zeta )\Big ) (t_{k+1}-\zeta )^{\xi -1} d\zeta \nonumber \\= & {} \frac{1-\xi }{ABC(\xi )} \Big (\phi I(t_k) - \vartheta R(t_k)\Big ) \nonumber \\{} & {} + \frac{\xi }{ABC(\xi ) \times \Gamma (\xi )} \sum _{\chi =0}^{k} \int _{t_\chi }^{t_{\chi +1}} \Big (\phi I(\zeta ) - \vartheta R(\zeta )\Big ) (t_{n+1}-\zeta )^{\xi -1} \end{aligned}$$

Within the interval $[t_\chi , t_{\chi +1}]$, the function $\Big (\phi I(\zeta ) - \vartheta R(\zeta )\Big )$, employing the two step lagrange polynomial interpolation, may be estimated like this:

48 $$\begin{aligned} P_\chi (\zeta )= & {} \frac{\zeta - t_{\chi -1}}{t_\chi - t_{\chi -1}} \Big (\phi I(t_\chi ) - \vartheta R(t_\chi )\Big ) - \frac{\zeta - t_{\chi }}{t_\chi - t_{\chi -1}} \Big (\phi I(t_{\chi -1}) - \vartheta R(t_{\chi -1})\Big ) \nonumber \\= & {} \frac{\Big (\phi I(t_\chi ) - \vartheta R(t_\chi )\Big )}{h} (\zeta -t_{\chi -1}) -\frac{\Big (\phi I(t_{\chi -1}) - \vartheta R(t_{\chi -1})\Big )}{h} (\zeta - t_{\chi }) \nonumber \\\simeq & {} \frac{\Big (\phi I(\chi ) - \vartheta R(\chi )\Big )}{h} (\zeta -t_{\chi -1}) -\frac{\Big (\phi I(\chi -1) - \vartheta R(\chi -1)\Big )}{h} (\zeta - t_{\chi }). \end{aligned}$$

Thus, the aforementioned estimate may be included in Eq. (47) in order to generate.

49 $$\begin{aligned} (R)_{k+1}= & {} (R)_0 + \frac{(1-\xi )}{ABC(\xi )} \Big (\phi I(t_k) - \vartheta R(t_k)\Big ) \nonumber \\{} & {} + \frac{\xi }{ABC(\xi ) \times \Gamma (\xi )}\sum _{\chi =0}^{k}\Bigg ( \frac{\Big (\phi I(\chi ) - \vartheta R(\chi )\Big )}{h} \int _{t_\chi }^{t_{\chi +1}} (\zeta - t_{\chi -1})(t_{k+1} - \zeta )^{\xi -1}d\zeta \nonumber \\{} & {} - \frac{\Big (\phi I(\chi -1) - \vartheta R(\chi -1)\Big )}{h} \int _{t_\chi }^{t_{\chi +1}} (\zeta - t_{\chi })(t_{k+1} - \zeta )^{\xi -1}d\zeta \Bigg ). \end{aligned}$$

For simplicity, we let

50 $$\begin{aligned} A_{\xi ,\chi ,1} = \int _{t_\chi }^{t_{\chi +1}} (\zeta - t_{\chi -1})(t_{k+1} - \zeta )^{\xi -1}d\zeta \end{aligned}$$

and also

51 $$\begin{aligned} A_{\xi ,\chi ,2}= & {} \int _{t_\chi }^{t_{\chi +1}} (\zeta - t_{\chi })(t_{k+1} - \zeta )^{\xi -1}d\zeta \nonumber \\ A_{\xi ,\chi ,1}= & {} \frac{(h^{\xi +1}) \{(k+1+\chi )^\xi (k-\chi +2+\xi ) - (k-\chi )^\xi (k-\chi +2+2\xi )\}}{\xi (\xi +1)}\nonumber \\ A_{\xi ,\chi ,2}= & {} \frac{(h^{\xi +1})\{(k+1+\chi )^{\xi +1} - (k-\chi )^\xi (k-\chi +1+\xi )\}}{\xi (\xi +1)}. \end{aligned}$$

By integrating Eqs. (50) and (51) and substituting them with Eq. (49), we can gain:

$$\begin{aligned} (R)_{k+1}= & {} (R)_0 + \frac{1-\xi }{ABC(\xi )} \Big (\phi I(t_k) - \vartheta R(t_k)\Big ) \\ {}&+ \frac{\xi }{ABC(\xi )} \sum _{\chi =0}^{k} \Bigg (\frac{h^\xi \Big (\phi I(\chi ) - \vartheta R(\chi )\Big )}{\Gamma (\xi +2)} ((k+1-\chi )^\xi (k-\chi +2+\xi ))\\{} & {} -\frac{h^\xi \Big (\phi I(\chi -1) - \vartheta R(\chi -1) \Big )}{\Gamma (\xi +2)} \Big ((k+1-\chi )^{\xi +1} - (k-\chi )^\xi (k-\chi +1+\xi )\Big ) \Bigg ). \end{aligned}$$

In the next portion which as follows, the numerical error of the aforementioned approximation is demonstrated in full detail.

## Error analysis

In this section, we will identify the inaccuracy that was created when we used our recommended method to approximate the fractional partial differential equation. In the next theorem, this will be proven.

### Theorem 5

Assuming that Eq. (7) has a nonlinear fractional, differential equations with the nonlocal and nonsingular kernel fractional derivative and that the second derivative of the function is monitored. It’s expected that the error can fulfill.

52 $$\begin{aligned} \left| R^\xi _k(S)\right|\le & {} \frac{\xi h^{\xi +2}}{2ABC(\xi )\Gamma (\xi +2)} \max _{[0, t_{k+1}]} \left| \frac{\partial ^2}{\partial \zeta ^2}[\varpropto -\mu \beta _{h}S(\zeta )E(\zeta )-\nu \beta _{e}S(\zeta )B(\zeta ) - \vartheta S(\zeta )] \right| \nonumber \\{} & {} \times ((k+1)^\xi - \xi k^\xi )\frac{k(k+4+2\xi )}{2} \end{aligned}$$

53 $$\begin{aligned} \left| R^\xi _k(E)\right|\le & {} \frac{\xi h^{\xi +2}}{2ABC(\xi )\Gamma (\xi +2)} \max _{[0, t_{k+1}]} \left| \frac{\partial ^2}{\partial \zeta ^2} [\mu \beta _{h} S(\zeta ) E(\zeta ) - \alpha E(\zeta ) - \vartheta E(\zeta )] \right| \nonumber \\{} & {} \times ((k+1)^\xi - \xi k^\xi ) \frac{k(k+4+2\xi )}{2} \end{aligned}$$

54 $$\begin{aligned} \left| R^\xi _k(I)\right|\le & {} \frac{\xi h^{\xi +2}}{2ABC(\xi )\Gamma (\xi +2)} \max _{[0, t_{k+1}]} \left| \frac{\partial ^2}{\partial \zeta ^2} [\alpha E(\zeta ) - \phi I(\zeta ) - \vartheta I(\zeta )] \right| \nonumber \\{} & {} \times ((k+1)^\xi - \xi n^\xi ) \frac{k(k+4+2\xi )}{2} \end{aligned}$$

55 $$\begin{aligned} \left| R^\xi _k(B)\right|\le & {} \frac{\xi h^{\xi +2}}{2ABC(\xi )\Gamma (\xi +2)} \max _{[0, t_{k+1}]} \left| \frac{\partial ^2}{\partial \zeta ^2} [\sigma I(\zeta ) + \nu \beta _{e} S(\zeta ) B(\zeta ) - \vartheta B(\zeta )] \right| \nonumber \\{} & {} \times ((k+1)^\xi - \xi k^\xi ) \frac{k(k+4+2\xi )}{2} \end{aligned}$$

56 $$\begin{aligned} \left| R^\xi _k(R)\right|\le & {} \frac{\xi h^{\xi +2}}{2ABC(\xi )\Gamma (\xi +2)} \max _{[0, t_{k+1}]} \left| \frac{\partial ^2}{\partial \zeta ^2} [\phi I(\zeta ) - \vartheta R(\zeta )] \right| \nonumber \\{} & {} \times ((k+1)^\xi - \xi k^\xi ) \frac{k(k+4+2\xi )}{2} \end{aligned}$$

### Proof

After the previous discussion on the numerical algorithm’s derivation, We’ve got:

57 $$\begin{aligned} S (t_{k+1}) - S(0)= & {} \frac{1-\xi }{ABC(\xi )} \big (\varpropto - \mu \beta _{h} S(t_k) E(t_k) - \nu \beta _{e} S(t_k) B(t_k) - \vartheta S(t_k)\big ) + \frac{\xi }{ABC(\xi )\Gamma (\xi )} \nonumber \\{} & {} \times \int _{0}^{t_{k+1}} \big (\varpropto - \mu \beta _{h} S(\zeta ) E(\zeta ) - \nu \beta _{e} S(\zeta ) B(\zeta ) - \vartheta S(\zeta )\big ) (t_{k+1} - \zeta )^{\xi -1}d\zeta \nonumber \\= & {} \frac{1-\xi }{ABC(\xi )} \big (\varpropto - \mu \beta _{h} S(t_k) E(t_k) - \nu \beta _{e} S(t_k) B(t_k) - \vartheta S(t_k)\big ) + \frac{\xi }{ABC(\xi )\Gamma (\xi )} \nonumber \\{} & {} \times \sum _{\chi =0}^{k}\int _{t_\chi }^{t_{\chi +1}} \big (\varpropto - \mu \beta _{h} S(\zeta ) E(\zeta ) - \nu \beta _{e} S(\zeta ) B(\zeta ) - \vartheta S(\zeta )\big ) (t_{k+1} - \zeta )^{\xi -1}d\zeta \nonumber \\= & {} \frac{1-\xi }{ABC(\xi )} \big (\varpropto - \mu \beta _{h} S(t_k) E(t_k) - \nu \beta _{e} S(t_k) B(t_k) - \vartheta S(t_k)\big ) + \frac{\xi }{ABC(\xi )\Gamma (\xi )}\nonumber \\{} & {} \times \sum _{\chi =0}^{k}\int _{t_\chi }^{t_{\chi +1}} \Big ( P_\chi (\zeta ) + \frac{(\zeta -t_\chi )(\zeta -t_{\chi -1})}{2!}\nonumber \\{} & {} \times \frac{\partial ^2}{\partial \zeta ^2} \Big [\varpropto - \mu \beta _{h} S(\zeta ) E(\zeta ) - \nu \beta _{e} S(\zeta ) B(\zeta ) - \vartheta S(\zeta )\Big ]_{\zeta =\varpi _\zeta } \Big ) (t_{k+1} - \zeta )^{\xi -1}d\zeta \nonumber \\= & {} \frac{1-\xi }{ABC(\xi )} \big (\varpropto - \mu \beta _{h} S(t_k) E(t_k) - \nu \beta _{e} S(t_k) B(t_k) - \vartheta S(t_k)\big ) + \frac{\xi }{ABC(\xi )\Gamma (\xi )}\nonumber \\{} & {} \sum _{\chi =0}^{k} \Bigg ( \frac{\big (\varpropto - \mu \beta _{h} S(\chi ) E(\chi ) - \nu \beta _{e} S(\chi ) B(\chi ) - \vartheta S(\chi )\big )}{h} \int _{t_\chi }^{t_{\chi +1}} (\zeta -t_{\chi -1})(t_{k+1}-\zeta )^{\xi -1}d\zeta \nonumber \\{} & {} -\frac{\big (\varpropto - \mu \beta _{h} S(\chi -1) E(\chi -1) - \nu \beta _{e} S(\chi -1) B(\chi -1) - \vartheta S(\chi -1)\big )}{h}\nonumber \\{} & {} \times \int _{t_\chi }^{t_{\chi +1}} (\zeta -t_\chi )(t_{k+1}-\zeta )^{\xi -1} d\zeta \Bigg )+ R^\xi _k(S), \end{aligned}$$

whereas the remainder,as a mentioned.

58 $$\begin{aligned} R^\xi _k(S)= & {} \frac{\xi }{ABC(\xi )\Gamma (\xi )} \sum _{\chi =0}^{k}\int _{t_\chi }^{t_{\chi +1}} \frac{\partial ^2}{\partial \zeta ^2}\Big [\varpropto - \mu \beta _{h} S(t_k) E(t_k) - \nu \beta _{e} S(t_k) B(t_k) - \nu \beta _{e} S(t_k) B(t_k) - \vartheta S(t_k)\Big ]_{\zeta =\varpi _\zeta } \nonumber \\{} & {} (t_{k+1} - \zeta )^{\xi -1}d\zeta . \end{aligned}$$

It is essential to remember that the function. $\zeta \ \underrightarrow{Analysis} \ (\zeta - t_{\chi -1})(t_{k+1}-\zeta )^{\xi -1}$ be positive with in the interval $[t_\chi , t_{\chi +1}]$, therefore there exists $\varpi _\zeta \in [t_\chi , t_{\chi +1}]$, such that

59 $$\begin{aligned} R^\xi _k(S)= & {} \frac{\xi }{ABC(\xi )\Gamma (\xi )} \sum _{\chi =0}^{k} \frac{\partial ^2}{\partial \zeta ^2}\Big [\varpropto - \mu \mu \beta _{h} S(\zeta ) E(\zeta ) - \nu \beta _{e} S(\zeta ) B(\zeta ) - \vartheta S(\zeta )\Big ]_{\zeta =\varpi _\chi } \frac{(\varpi _\chi -t_\chi )}{2} \nonumber \\{} & {} \times \int _{t_\chi }^{t_{\chi +1}} (\zeta -t_{\chi -1}) (t_{k+1} - \zeta )^{\xi -1}d\zeta \nonumber \\= & {} \frac{\xi }{ABC(\xi )\Gamma (\xi )} \sum _{\chi =0}^{k} \frac{\partial ^2}{\partial \zeta ^2}\Big [\varpropto - \mu \beta _{h} S(\zeta ) E(\zeta ) - \nu \beta _{e} S(\zeta ) B(\zeta ) - \vartheta S(\zeta )\Big ]_{\zeta =\varpi _\chi } \frac{(\varpi _\chi -t_\chi )}{2}\nonumber \\{} & {} \times A_{\xi ,\chi ,1} h^{\xi +1}. \end{aligned}$$

Accordingly, employing the norm characteristics and employing the norm on both sides, we now have:

60 $$\begin{aligned} A_{\xi ,\chi ,1}= & {} \frac{(k+1-\chi )^\xi (k-\chi +2+\xi )-(k-\chi )^\xi (k-\chi +2+2\xi )}{\xi (\xi +1)},\nonumber \\ |R^\xi _k(S)|\le & {} \frac{\xi h^{\xi +2}}{2 ABC(\xi )\Gamma (\xi +2)} \max _{[0, t_{k+1}]} \left| \sum _{\chi =0}^{k} \frac{\partial ^2}{\partial \zeta ^2}\Big [\varpropto - \mu \beta _{h} S(\zeta ) E(\zeta ) - \nu \beta _{e} S(\zeta ) B(\zeta ) - \vartheta S(\zeta )\Big ] \right| \nonumber \\{} & {} \left| \sum _{\chi =0}^{k} (((k+1-\chi )^\xi (k-\chi +2+\xi )-(k-\chi )^\xi (k-\chi +2+2\xi ))) \right| . \end{aligned}$$

The right-side summation converges in the manner shown below:

61 $$\begin{aligned}{} & {} (((k+1-\chi )^\xi (k-\chi +2+\xi )-(k-\chi )^\xi (k-\chi +2+2\xi ))) \nonumber \\{} & {} \quad =(((k+1-\chi )^\xi (k-\chi +2+\xi )-(k-\chi )^\xi (k-\chi +2+\xi +\xi )))\nonumber \\{} & {} \quad = (k-\chi +2+\xi )((k+1-\chi )^\xi - \xi (k-\chi )^\xi ) \nonumber \\{} & {} \qquad ((k+1-\chi )^\xi - \xi (k-\chi )^\xi ) \le ((k+1)^\xi - \xi k^\xi ) \nonumber \\{} & {} \qquad \sum _{\chi =0}^{k} (k-\chi +2+\xi ) = \frac{k(k+4+2\xi )}{2}. \end{aligned}$$

Thus

62 $$\begin{aligned} |R^\xi _k( S)|\le & {} \frac{\xi h^{\xi +2}}{2 ABC(\xi )\Gamma (\xi +2)} \max _{[0, t_{k+1}]} \left| \sum _{\chi =0}^{k} \frac{\partial ^2}{\partial \zeta ^2}\Big [\varpropto - \mu \beta _{h} S(\zeta ) E(\zeta ) - \nu \beta _{e} S(\zeta ) B(\zeta ) - \vartheta S(\zeta )\Big ] \right| \nonumber \\{} & {} ((k+1)^\xi - \xi k^\xi )\frac{k(k+4+2\xi )}{2}. \end{aligned}$$

Similarly for *E* of (7), we solve

63 $$\begin{aligned} E (t_{k+1}) - E(0)= & {} \frac{1-\xi }{ABC(\xi )} \big (\mu \beta _{h} S(t_k) E(t_k) - \alpha E(t_k) - \vartheta E(t_k)\big ) + \frac{\xi }{ABC(\xi )\Gamma (\xi )}\nonumber \\{} & {} \times \int _{0}^{t_{k+1}} \big (\mu \beta _{h} S(\zeta ) E(\zeta ) - \alpha E(\zeta ) - \vartheta E(\zeta )\big ) (t_{n+1} - \zeta )^{\xi -1}d\zeta \nonumber \\= & {} \frac{1-\xi }{ABC(\xi )} \big (\mu \beta _{h} S(t_k) E(t_k) - \alpha E(t_k) - \vartheta E(t_k)\big ) + \frac{\xi }{ABC(\xi )\Gamma (\xi )}\nonumber \\{} & {} \times \sum _{\chi =0}^{k}\int _{t_\chi }^{t_{\chi +1}} \big (\mu \beta _{h} S(\zeta ) E(\zeta ) - \alpha E(\zeta ) - \vartheta (t_{k+1} - (t_{k+1} - \zeta )^{\xi -1}d\zeta \nonumber \\= & {} \frac{1-\xi }{ABC(\xi )} \big (\mu \beta _{h} S(t_k) E(t_k) - \alpha E(t_k) - \vartheta E(t_k)\big ) + \frac{\xi }{ABC(\xi )\Gamma (\xi )}\nonumber \\{} & {} \times \sum _{\chi =0}^{k}\int _{t_\chi }^{t_{\chi +1}} \Big ( P_\chi (\zeta ) + \frac{(\zeta -t_\chi )(\zeta -t_{\chi -1})}{2!}\nonumber \\{} & {} \times \frac{\partial ^2}{\partial \zeta ^2}\Big [\mu \beta _{h} S(\zeta ) E(\zeta ) - \alpha E(\zeta ) - \vartheta E(\zeta )\Big ]_{\zeta =\varpi _\zeta } \Big ) (t_{k+1} - \zeta )^{\xi -1}d\zeta . \end{aligned}$$

64 $$\begin{aligned} E (t_{k+1}) - E(0)= & {} \frac{1-\xi }{ABC(\xi )} \big (\mu \beta _{h} S(t_k) E(t_k) - \alpha E(t_k) - \vartheta E(t_k)\big ) + \frac{\xi }{ABC(\xi )\Gamma (\xi )}\nonumber \\{} & {} \times \sum _{\chi =0}^{k} \Bigg ( \frac{\big (\mu \beta _{h} S(\chi ) E(\chi ) - \alpha E(\chi ) - \vartheta E(\chi )\big )}{h} \int _{t_\chi }^{t_{\chi +1}} (\zeta -t_{\chi -1})(t_{k+1}-\zeta )^{\xi -1}d\zeta \nonumber \\{} & {} -\frac{\big (\mu \beta _{h} S(\chi -1) E(\chi -1) - \alpha E(\chi -1) - \vartheta E(\chi -1)\big )}{h} \nonumber \\{} & {} \times \int _{t_\chi }^{t_{\chi +1}} (\zeta -t_\chi )(t_{k+1}-\zeta )^{\xi -1}d\zeta \Bigg )+ R^\xi _k(E) \end{aligned}$$

whereas the remainder, as a mentioned.

65 $$\begin{aligned} R^\xi _k(E)= & {} \frac{\xi }{ABC(\xi )\Gamma (\xi )} \sum _{\chi =0}^{k}\int _{t_\chi }^{t_{\chi +1}} \frac{(\zeta -t_\chi )(\zeta -t_{\chi -1})}{2!}\nonumber \\{} & {} \times \frac{\partial ^2}{\partial \zeta ^2}\Big [\mu \beta _{h} S(\zeta ) E(\zeta ) - E(\zeta ) - \vartheta E(\zeta )\Big ]_{\zeta =\varpi _\zeta } (t_{k+1} - \zeta )^{\xi -1}d\zeta . \end{aligned}$$

It is essential to remember that the function. $\zeta \ \underrightarrow{Analysis} \ (\zeta - t_{\chi -1})(t_{k+1}-\zeta )^{\xi -1}$ be positive with in the interval $[t_\chi , t_{\chi +1}]$, therefore there exists $\varpi _\zeta \in [t_\chi , t_{\chi +1}]$, such that

66 $$\begin{aligned} R^\xi _n(E)= & {} \frac{\xi }{ABC(\xi )\Gamma (\xi )} \sum _{\chi =0}^{k} \frac{\partial ^2}{\partial \zeta ^2}\Big [\mu \beta _{h} S(\zeta ) E(\zeta ) - \alpha E(\zeta ) - \vartheta E(\zeta )]_{\zeta =\varpi _\chi } \frac{(\varpi _\chi -t_\chi )}{2}\nonumber \\{} & {} \times \int _{t_\chi }^{t_{\chi +1}} (\zeta -t_{\chi -1}) (t_{k+1} - \zeta )^{\xi 
-1}d\zeta \nonumber \\= & {} \frac{\xi }{ABC(\xi )\Gamma (\xi )} \sum _{\chi =0}^{k} \frac{\partial ^2}{\partial \zeta ^2}\Big [\mu \beta _{h} S(\zeta ) E(\zeta ) - \alpha E(\zeta ) - \vartheta E(\zeta )\Big ]_{\zeta =\varpi _\chi } \frac{(\varpi _\chi -t_\chi )}{2} \times A_{\xi ,\chi ,1} h^{\xi +1}. \end{aligned}$$

Accordingly, employing the norm characteristics and employing the norm on both sides, we now have:

67 $$\begin{aligned} A_{\xi ,\chi ,1}= & {} \frac{(k+1-\chi )^\xi (k-\chi +2+\xi )-(k-\chi )^\xi (k-\chi +2+2\xi )}{\xi (\xi +1)},\nonumber \\ |R^\xi _k(E)|\le & {} \frac{\xi h^{\xi +2}}{2 ABC(\xi )\Gamma (\xi +2)} \max _{[0, t_{k+1}]} \Bigg | \sum _{\chi =0}^{k} \frac{\partial ^2}{\partial \zeta ^2}\big [\mu \beta _{h} S(\zeta ) E(\zeta ) - \alpha E(\zeta ) - \vartheta E(\zeta )\big ] \Bigg | \nonumber \\{} & {} \left| \sum _{\chi =0}^{k} (((k+1-\chi )^\xi (k-\chi +2+\xi )-(k-\chi )^\xi (k-\chi +2+2\xi ))) \right| . \end{aligned}$$

The right-side summation converges in the manner shown below:

68 $$\begin{aligned}{} & {} (((k+1-\chi )^\xi (k-\chi +2+\xi )-(k-\chi )^\xi (k-\chi +2+2\xi ))) \nonumber \\{} & {} \quad =(((k+1-\chi )^\xi (k-\chi +2+\xi )-(k-\chi )^\xi (k-\chi +2+\xi +\xi ))) \nonumber \\{} & {} \quad = (k-\chi +2+\xi )((k+1-\chi )^\xi - \xi (k-\chi )^\xi ) \nonumber \\{} & {} \qquad ((k+1-\chi )^\xi - \xi (k-\chi )^\xi ) \le ((k+1)^\xi - \xi n^\xi ) \nonumber \\{} & {} \qquad \sum _{\chi =0}^{k} (k-\chi +2+\xi ) = \frac{k(k+4+2\xi )}{2}. \end{aligned}$$

Thus

69 $$\begin{aligned} |R^\xi _k(E)|\le & {} \frac{\xi h^{\xi +2}}{2 ABC(\xi )\Gamma (\xi +2)} \max _{[0, t_{k+1}]} \Bigg | \sum _{\chi =0}^{k} \frac{\partial ^2}{\partial \zeta ^2}\big [\mu \beta _{h} S(\zeta ) E(\zeta ) - \alpha E(\zeta ) - \vartheta E(\zeta )\big ] \Bigg |\nonumber \\{} & {} \times ((k+1)^\xi - \xi k^\xi )\frac{k(k+4+2\xi )}{2}. \end{aligned}$$

Next, we solve error approximation of *I* of (7), and get

70 $$\begin{aligned} I (t_{k+1}) - I(0)= & {} \frac{1-\xi }{ABC(\xi )} \big (\alpha E(t_k) - \phi I(t_k) - \vartheta I(t_k)\big ) \nonumber \\{} & {} +\frac{\xi }{ABC(\xi )\Gamma (\xi )} \int _{0}^{t_{k+1}} \big (\alpha E(\zeta ) - \phi I(\zeta ) - \vartheta I(\zeta )\big ) (t_{k+1} - \zeta )^{\xi -1}d\zeta \nonumber \\= & {} \frac{1-\xi }{ABC(\xi )} \big (\alpha E(t_k) - \phi I(t_k) - \vartheta I(t_k)\big ) \nonumber \\{} & {} +\frac{\xi }{ABC(\xi )\Gamma (\xi )}\sum _{\chi =0}^{k}\int _{t_\chi }^{t_{\chi +1}} \big (\alpha E(\zeta ) - \phi I(\zeta ) - \vartheta I(\zeta )\big ) (t_{k+1} - \zeta )^{\xi -1}d\zeta \nonumber \\= & {} \frac{1-\xi }{ABC(\xi )} \big (\alpha E(t_k) - \phi I(t_k) - \vartheta I(t_k)\big ) + \frac{\xi }{ABC(\xi )\Gamma (\xi )}\nonumber \\{} & {} \times \sum _{\chi =0}^{k}\int _{t_\chi }^{t_{\chi +1}} \left( P_\chi (\zeta ) + \frac{(\zeta -t_\chi )(\zeta -t_{\chi -1})}{2!} \frac{\partial ^2}{\partial \zeta ^2}\Big [\alpha E(t) - \phi I(t) - \vartheta I(t)\Big ]_{\zeta =\varpi _\zeta } \right) (t_{k+1} - \zeta )^{\xi -1}d\zeta \nonumber \\ I (t_{k+1}) - I(0)= & {} \frac{1-\xi }{ABC(\xi )} \big (\alpha E(t_k) - \phi I(t_k) - \vartheta I(t_k)\big ) \nonumber \\{} & {} +\frac{\xi }{ABC(\xi )\Gamma (\xi )}\sum _{\chi =0}^{k} \Bigg ( \frac{\big (\alpha E(\chi ) - \phi I(\chi ) - \vartheta I(\chi )\big )}{h} \nonumber \\{} & {} \times \int _{t_\chi }^{t_{\chi +1}} (\zeta -t_{\chi -1})(t_{k+1}-\zeta )^{\xi -1}d\zeta \nonumber \\{} & {} -\frac{\big (\alpha E(\chi -1) - \phi I(\chi -1) - \vartheta I(\chi -1)\big )}{h} \int _{t_\chi }^{t_{\chi +1}} (\zeta -t_\chi )(t_{k+1}-\zeta )^{\xi -1}d\zeta \Bigg )+ R^\xi _k(I), \end{aligned}$$

whereas the remainder, as a mentioned.

71 $$\begin{aligned} R^\xi _k(I) = \frac{\xi }{ABC(\xi )\Gamma (\xi )} \sum _{\chi =0}^{k}\int _{t_\chi }^{t_{\chi +1}} \frac{\partial ^2}{\partial \zeta ^2}\Big [\alpha E(\zeta ) - \phi I(\zeta ) - \vartheta I(\zeta )\Big ]_{\zeta =\varpi _\zeta } I(\zeta )\Big ]_{\zeta =\varpi _\zeta } (t_{k+1} - \zeta )^{\xi -1}d\zeta . \end{aligned}$$

It is essential to remember that the function. $\zeta \ \underrightarrow{Analysis} \ (\zeta - t_{\chi -1})(t_{k+1}-\zeta )^{\xi -1}$ be positive with in the interval $[t_\chi , t_{\chi +1}]$, therefore there exists $\varpi _\zeta \in [t_\chi , t_{\chi +1}]$, such that

72 $$\begin{aligned} R^\xi _k(I)= & {} \frac{\xi }{ABC(\xi )\Gamma (\xi )} \sum _{\chi =0}^{k} \frac{\partial ^2}{\partial \zeta ^2}\Big [\alpha E(\zeta ) - \phi I(\zeta ) - \vartheta I(\zeta )\Big ]_{\zeta =\varpi _\chi } \frac{(\varpi _\chi -t_\chi )}{2} \int _{t_\chi }^{t_{\chi +1}} (\zeta -t_{\chi -1}) (t_{k+1} - \zeta )^{\xi -1}d\zeta \nonumber \\= & {} \frac{\xi }{ABC(\xi )\Gamma (\xi )} \sum _{\chi =0}^{k} \frac{\partial ^2}{\partial \zeta ^2}\Big [\alpha E(\zeta ) - \phi I(\zeta ) - \vartheta I(\zeta )\Big ]_{\zeta =\varpi _\chi } \frac{(\varpi _\chi -t_\chi )}{2} \times A_{\xi ,\chi ,1} h^{\xi +1}. \end{aligned}$$

Accordingly, employing the norm characteristics and employing the norm on both sides, we now have:

73 $$\begin{aligned} A_{\xi ,k,1}= & {} \frac{(k+1-\chi )^\xi (k-\chi +2+\xi )-(k-k)^\xi (k-\chi +2+2\xi )}{\xi (\xi +1)},\nonumber \\ |R^\xi _k(I)|\le & {} \frac{\xi h^{\xi +2}}{2 ABC(\xi )\Gamma (\xi +2)} \max _{[0, t_{k+1}]} \Bigg | \sum _{\chi =0}^{k} \frac{\partial ^2}{\partial \zeta ^2}\Big [\alpha E(\zeta ) - \phi I(\zeta ) - \vartheta I(\zeta )\Big ] \Bigg | \nonumber \\{} & {} \left| \sum _{\chi =0}^{k} (((k+1-\chi )^\xi (k-\chi +2+\xi )-(k-\chi )^\xi (k-\chi +2+2\xi ))) \right| . \end{aligned}$$

The right-side summation converges in the manner shown below:

74 $$\begin{aligned}{} & {} (((k+1-\chi )^\xi (k-\chi +2+\xi )-(k-\chi )^\xi (k-\chi +2+2\xi ))) \nonumber \\{} & {} \quad = (((k+1-\chi )^\xi (k-\chi +2+\xi )-(k-\chi )^\xi (k-\chi +2+\xi +\xi ))) \nonumber \\{} & {} \quad = (k-\chi +2+\xi )((k+1-\chi )^\xi - \xi (k-\chi )^\xi ) \nonumber \\{} & {} \qquad ((k+1-\chi )^\xi - \xi (k-\chi )^\xi ) \le ((k+1)^\xi - \xi k^\xi ) \nonumber \\{} & {} \qquad \sum _{\chi =0}^{k} (k-\chi +2+\xi ) = \frac{k(k+4+2\xi )}{2}. \end{aligned}$$

Thus

75 $$\begin{aligned} |R^\xi _k(I)|\le & {} \frac{\xi h^{\xi +2}}{2 ABC(\xi )\Gamma (\xi +2)} \max _{[0, t_{k+1}]} \Bigg | \sum _{\chi =0}^{k} \frac{\partial ^2}{\partial \zeta ^2}\Big [\alpha E(\zeta ) - \phi I(\zeta ) - \vartheta I(\zeta )\Big ] \Bigg | \nonumber \\{} & {} ((k+1)^\xi - \xi k^\xi )\frac{k(k+4+2\xi )}{2}. \end{aligned}$$

Now for *B* of (7) solve error analysis, we get

76 $$\begin{aligned} B (t_{k+1}) - B(0)= & {} \frac{1-\xi }{ABC(\xi )} \big (\sigma I(t_k) + \nu \beta _{e} S(t_k) B(t_k) - \vartheta B(t_k)\big ) + \frac{\xi }{ABC(\xi )\Gamma (\xi )} \nonumber \\{} & {} \times \int _{0}^{t_{k+1}} \big (\sigma I(\zeta ) + \nu \beta _{e} S(\zeta ) B(\zeta ) - \vartheta B(\zeta )\big ) (t_{k+1} - \zeta )^{\xi -1}d\zeta \nonumber \\= & {} \frac{1-\xi }{ABC(\xi )} \big (\sigma I(t_k) + \nu \beta _{e} S(t_k) B(t_k) - \vartheta B(t_k)\big ) + \frac{\xi }{ABC(\xi )\Gamma (\xi )}\nonumber \\{} & {} \times \sum _{\chi =0}^{k}\int _{t_\chi }^{t_{\chi +1}} \big (\sigma I(\zeta ) + \nu \beta _{e} S(\zeta ) B(\zeta ) - \vartheta (t_{k+1} - (t_{k+1} - \zeta )^{\xi -1}d\zeta \nonumber \\= & {} \frac{1-\xi }{ABC(\xi )} \big (\sigma I(t_k) + \nu \beta _{e} S(t_k) B(t_k) - \vartheta B(t_k)\big ) + \frac{\xi }{ABC(\xi )\Gamma (\xi )}\nonumber \\{} & {} \times \sum _{\chi =0}^{k}\int _{t_\chi }^{t_{\chi +1}} \left( P_\chi (\zeta ) + \frac{(\zeta -t_\chi )(\zeta -t_{\chi -1})}{2!} \frac{\partial ^2}{\partial \zeta ^2}\Big [\sigma I(\zeta ) + \nu \beta _{e} S(\zeta ) B(\zeta ) - \vartheta B(\zeta )\Big ]_{\zeta =\varpi _\zeta } \right) \nonumber \\{} & {} \times (t_{k+1} - \zeta )^{\xi -1}d\zeta \nonumber \\ B (t_{k+1}) - B(0)= & {} \frac{1-\xi }{ABC(\xi )} \big (\sigma I(t_k) + \nu \beta _{e} S(t_k) B(t_k) - \vartheta B(t_k)\big ) + \frac{\xi }{ABC(\xi )\Gamma (\xi )}\nonumber \\{} & {} \sum _{\chi =0}^{k} \Bigg ( \frac{\big (\sigma I(\chi ) + \nu \beta _{e} S(\chi ) B(\chi ) - \vartheta B(\chi )\big )}{h} \int _{t_\chi }^{t_{\chi +1}} (\zeta -t_{\chi -1})(t_{k+1}-\zeta )^{\xi -1}d\zeta -\nonumber \\{} & {} \frac{\big (\sigma I(\chi -1) + \nu \beta _{e} S(\chi -1) B(\chi -1) - \vartheta B(\chi -1)\big )}{h}\nonumber \\{} & {} \times \int _{t_\chi }^{t_{\chi +1}} (\zeta -t_\chi )(t_{k+1}-\zeta )^{\xi -1}d\zeta \Bigg ) + R^\xi _k(B), \end{aligned}$$

$$\square $$

whereas the remainder, as a mentioned.

77 $$\begin{aligned} R^\xi _k(B) = \frac{\xi }{ABC(\xi )\Gamma (\xi )} \sum _{\chi =0}^{k}\int _{t_\chi }^{t_{\chi +1}} \frac{\partial ^2}{\partial \zeta ^2}\Big [\sigma I(\zeta ) + \nu \beta _{e} S(\zeta ) B(\zeta ) - \vartheta B(\zeta ) - \vartheta B(\zeta )\Big ]_{\zeta =\varpi _\zeta } (t_{k+1} - \zeta )^{\xi -1}d\zeta . \end{aligned}$$

It is essential to remember that the function. $\zeta \ \underrightarrow{Analysis} \ (\zeta - t_{\chi -1})(t_{k+1}-\zeta )^{\xi -1}$ be positive with in the interval $[t_\chi , t_{\chi +1}]$, therefore there exists $\varpi _\zeta \in [t_\chi , t_{\chi +1}]$, such that

78 $$\begin{aligned} R^\xi _k(B)= & {} \frac{\xi }{ABC(\xi )\Gamma (\xi )} \sum _{\chi =0}^{k} \frac{\partial ^2}{\partial \zeta ^2}\Big [\sigma I(\zeta ) + \nu \beta _{e} S(\zeta ) B(\zeta ) - \vartheta B(\zeta )\Big ]_{\zeta =\varpi _\chi } \frac{(\varpi _\chi -t_\chi )}{2}\nonumber \\{} & {} \times \int _{t_\chi }^{t_{\chi +1}} (\zeta -t_{\chi -1}) (t_{k+1} - \zeta )^{\xi -1}d\zeta \nonumber \\= & {} \frac{\xi }{ABC(\xi )\Gamma (\xi )} \sum _{\chi =0}^{k} \frac{\partial ^2}{\partial \zeta ^2}\Big [\sigma I(\zeta ) + \nu \beta _{e} S(\zeta ) B(\zeta ) - \vartheta B(\zeta )\Big ]_{\zeta =\varpi _\chi } \frac{(\varpi _\chi -t_\chi )}{2} \times A_{\xi ,\chi ,1} h^{\xi +1}. \end{aligned}$$

Accordingly, employing the norm characteristics and employing the norm on both sides, we now have:

79 $$\begin{aligned} A_{\xi ,\chi ,1}= & {} \frac{(k+1-\chi )^\xi (k-\chi +2+\xi )-(k-\chi )^\xi (k-\chi +2+2\xi )}{\xi (\xi +1)},\nonumber \\ |R^\xi _k(B)|\le & {} \frac{\xi h^{\xi +2}}{2 ABC(\xi )\Gamma (\xi +2)} \max _{[0, t_{k+1}]} \left| \sum _{\chi =0}^{k} \frac{\partial ^2}{\partial \zeta ^2}\Big [\sigma I(\zeta ) + \nu \beta _{e} S(\zeta ) B(\zeta ) - \vartheta B(\zeta )\Big ] \right| \nonumber \\{} & {} \times \left| \sum _{\chi =0}^{k} (((k+1-\chi )^\xi (k-\chi +2+\xi )-(k-\chi )^\xi (k-\chi +2+2\xi ))) \right| . \end{aligned}$$

The right-side summation converges in the manner shown below:

80 $$\begin{aligned}{} & {} (((k+1-\chi )^\xi (k-\chi +2+\xi )-(k-\chi )^\xi (k-\chi +2+2\xi ))) \nonumber \\{} & {} \quad = (((k+1-\chi )^\xi (k-\chi +2+\xi )-(k-\chi )^\xi (k-\chi +2+\xi +\xi ))) \nonumber \\{} & {} \quad = (k-\chi +2+\xi )((k+1-\chi )^\xi - \xi (k-\chi )^\xi ) \nonumber \\{} & {} \qquad ((k+1-\chi )^\xi - \xi (k-\chi )^\xi ) \le ((k+1)^\xi - \xi k^\xi ) \nonumber \\{} & {} \qquad \sum _{\chi =0}^{k} (k-\chi +2+\xi ) = \frac{k(k+4+2\xi )}{2}. \end{aligned}$$

Thus

81 $$\begin{aligned} |R^\xi _k(B)|\le & {} \frac{\xi h^{\xi +2}}{2 ABC(\xi )\Gamma (\xi +2)} \max _{[0, t_{k+1}]} \left| \sum _{\chi =0}^{k} \frac{\partial ^2}{\partial \zeta ^2}\Big [\sigma I(\zeta ) + \nu \beta _{e} S(\zeta ) B(\zeta ) - \vartheta B(\zeta )\Big ] \right| \nonumber \\{} & {} \times ((k+1)^\xi - \xi n^\xi )\frac{k(k+4+2\xi )}{2}. \end{aligned}$$

Similarly for *R* of (7), we solve

82 $$\begin{aligned} R (t_{k+1}) - R(0)= & {} \frac{1-\xi }{ABC(\xi )} \big (\phi I(t_k) - \vartheta R(t_k)\big ) + \frac{\xi }{ABC(\xi )\Gamma (\xi )} \int _{0}^{t_{k+1}} \big (\phi I(\zeta ) - \vartheta R(\zeta )\big ) (t_{k+1} - \zeta )^{\xi -1}d\zeta \nonumber \\= & {} \frac{1-\xi }{ABC(\xi )} \big (\phi I(t_k) - \vartheta R(t_k)\big ) + \frac{\xi }{ABC(\xi )\Gamma (\xi )} \sum _{\chi =0}^{k}\int _{t_\chi }^{t_{\chi +1}} \big (\phi I(\zeta ) - \vartheta R(\zeta )\big ) (t_{k+1} - \nonumber \\= & {} \frac{1-\xi }{ABC(\xi )} \big (\phi I(t_k) - \vartheta R(t_k)\big ) + \frac{\xi }{ABC(\xi )\Gamma (\xi )}\nonumber \\{} & {} \times \sum _{\chi =0}^{k}\int _{t_\chi }^{t_{\chi +1}} \left( P_\chi (\zeta ) + \frac{(\zeta -t_\chi )(\zeta -t_{\chi -1})}{2!} \frac{\partial ^2}{\partial \zeta ^2}\Big [\phi I(\zeta ) - \vartheta R(\zeta )\Big ]_{\zeta =\varpi _\zeta } \right) \nonumber \\{} & {} \times (t_{k+1} - \zeta )^{\xi -1}d\zeta \nonumber \\ R (t_{k+1}) - R(0)= & {} \frac{1-\xi }{ABC(\xi )} \big (\phi I(t_k) - \vartheta R(t_k)\big ) + \frac{\xi }{ABC(\xi )\Gamma (\xi )}\nonumber \\{} & {} \times \sum _{\chi =0}^{k} \Bigg ( \frac{\big (\phi I(\chi ) - \vartheta R(\chi )\big )}{h} \int _{t_\chi }^{t_{\chi +1}} (\zeta -t_{\chi -1})(t_{k+1}-\zeta )^{\xi -1}d\zeta -\nonumber \\ {}{} & {} \times \frac{\big (\phi I(\chi -1) - \vartheta R(\chi -1)\big )}{h} \int _{t_\chi }^{t_{\chi +1}} (\zeta -t_\chi )(t_{k+1}-\zeta )^{\xi -1}d\zeta \Bigg ) + R^\xi _k(R), \end{aligned}$$

whereas the remainder, as a mentioned.

83 $$\begin{aligned} R^\xi _k(R) = \frac{\xi }{ABC(\xi )\Gamma (\xi )} \sum _{\chi =0}^{k}\int _{t_\chi }^{t_{\chi +1}} \frac{(\zeta -t_\chi )(\zeta -t_{\chi -1})}{2!} \frac{\partial ^2}{\partial \zeta ^2}\Big [\phi I(\zeta ) - \vartheta \zeta )^{\xi -1}d\zeta . \end{aligned}$$

It is essential to remember that the function. $\zeta \ \underrightarrow{Analysis} \ (\zeta - t_{\chi -1})(t_{k+1}-\zeta )^{\xi -1}$ be positive with in the interval $[t_\chi , t_{\chi +1}]$, therefore there exists $\varpi _\zeta \in [t_\chi , t_{\chi +1}]$, such that

84 $$\begin{aligned} R^\xi _k(R)= & {} \frac{\xi }{ABC(\xi )\Gamma (\xi )} \sum _{\chi =0}^{k} \frac{\partial ^2}{\partial \zeta ^2}\Big [\phi I(\zeta ) - \vartheta R(\zeta )\Big ]_{\zeta =\varpi _\chi } \frac{(\varpi _\chi -t_\chi )}{2} \nonumber \\{} & {} \times \int _{t_\chi }^{t_{\chi +1}} (\zeta -t_{\chi -1}) (t_{k+1} - \zeta )^{\xi -1}d\zeta \nonumber \\= & {} \frac{\xi }{ABC(\xi )\Gamma (\xi )} \sum _{\chi =0}^{k} \frac{\partial ^2}{\partial \zeta ^2}\Big [\phi I(\zeta ) - \vartheta R(\zeta )\Big ]_{\zeta =\varpi _\chi } \frac{(\varpi _\chi -t_\chi )}{2} \times A_{\xi ,\chi ,1} h^{\xi +1}. \end{aligned}$$

Accordingly, employing the norm characteristics and employing the norm on both sides, we now have:

85 $$\begin{aligned} A_{\xi ,\chi ,1}= & {} \frac{(k+1-\chi )^\xi (k-\chi +2+\xi )-(k-\chi )^\xi (k-\chi +2+2\xi )}{\xi (\xi +1)},\nonumber \\ |R^\xi _k(R)|\le & {} \frac{\xi h^{\xi +2}}{2 ABC(\xi )\Gamma (\xi +2)} \max _{[0, t_{k+1}]} \left| \sum _{\chi =0}^{k} \frac{\partial ^2}{\partial \zeta ^2}\Big [\big (\phi I(\zeta ) - \vartheta R(\zeta )\big )\Big ] \right| \nonumber \\{} & {} \times \left| \sum _{\chi =0}^{k} (((k+1-\chi )^\xi (k-\chi +2+\xi )-(k-\chi )^\xi (k-\chi +2+2\xi ))) \right| . \end{aligned}$$

The right-side summation converges in the manner shown below:

86 $$\begin{aligned}{} & {} (((k+1-\chi )^\xi (k-\chi +2+\xi )-(k-\chi )^\xi (k-\chi +2+2\xi ))) \nonumber \\{} & {} \quad = (((k+1-\chi )^\xi (k-\chi +2+\xi )-(k-\chi )^\xi (k-\chi +2+\xi +\xi ))) \nonumber \\{} & {} \quad = (k-\chi +2+\xi )((k+1-\chi )^\xi - \xi (k-\chi )^\xi ) \nonumber \\{} & {} \qquad ((k+1-\chi )^\xi - \xi (k-\chi )^\xi ) \le ((k+1)^\xi - \xi n^\xi ) \nonumber \\{} & {} \qquad \sum _{\chi =0}^{k} (k-\chi +2+\xi ) = \frac{k(k+4+2\xi )}{2}. \end{aligned}$$

Thus

87 $$\begin{aligned} |R^\xi _k(R)|\le & {} \frac{\xi h^{\xi +2}}{2 ABC(\xi )\Gamma (\xi +2)} \max _{[0, t_{k+1}]} \left| \sum _{\chi =0}^{k} \frac{\partial ^2}{\partial \zeta ^2}\Big [\phi I(\zeta ) - \vartheta R(\zeta )\Big ] \right| ((k+1)^\xi - \xi k^\xi )\frac{k(k+4+2\xi )}{2}. \end{aligned}$$

## Simulation explantation

**Figure 7:**
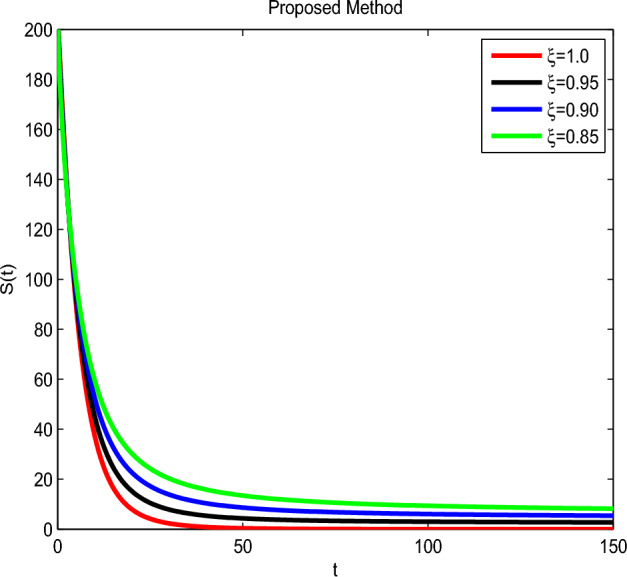
*S*(*t*) employing Atangana–Baleanu in Caputo sense (ABC) fractional operator for $R_0 < 1$.

This section explains how to statistically simulate the ABC technique for the cholera model with the influence of both asymptomatic and symptomatic transmission, which is the suggested technique. We used the ABC operator in combination with fractal-fractional derivative of cholera model under predefined beginning circumstances to analyze the disease transmission through simulations involving both symptomatic and asymptomatic transmission. Fractional values can be employed for determining a nonlinear system. Figures 3, 4, 5, 6, 7 show the simulation data with various fractional order values.

**Figure 8:**
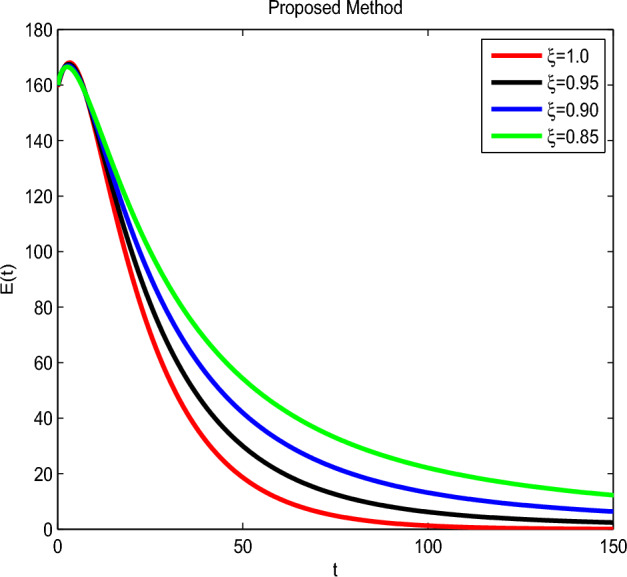
*E*(*t*) employing Atangana–Baleanu in Caputo sense (ABC) fractional operator for $R_0 < 1$.

**Figure 9:**
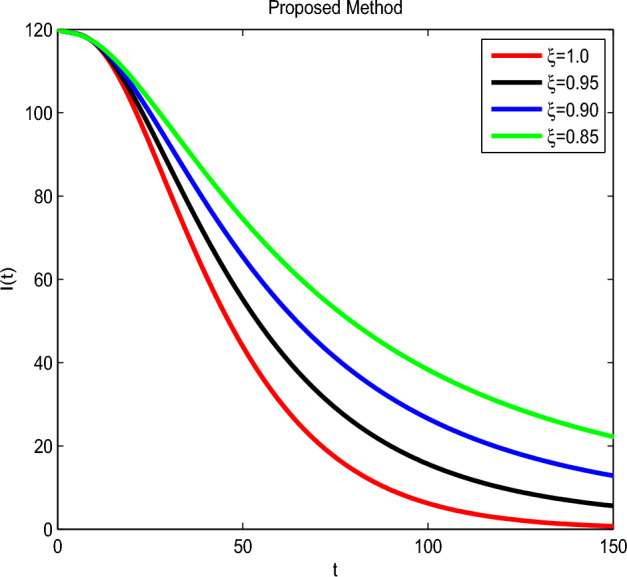
*I*(*t*) employing Atangana–Baleanu in Caputo sense (ABC) fractional operator for $R_0 < 1$.

**Figure 10:**
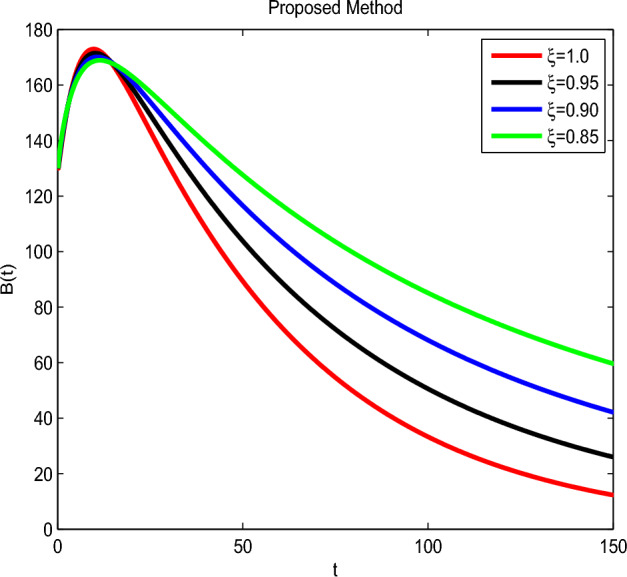
*B*(*t*) employing Atangana–Baleanu in Caputo sense (ABC) fractional operator for $R_0 < 1$.

**Figure 11:**
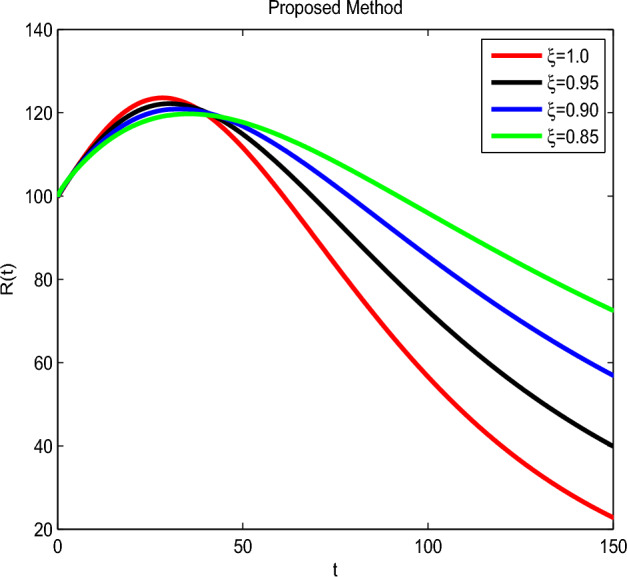
*R*(*t*) employing Atangana–Baleanu in Caputo sense (ABC) fractional operator for $R_0 < 1$.

The following examples demonstrate the efficacy of the achieved theoretical consequences. Utilizing non-integer parametric choices for cholera illness, considering both symptomatic and asymptomatic transmission, yields reliable results. MATLAB coding is employed to develop a computer simulation for the fractional-order cholera model, considering both symptomatic and asymptomatic transmission. The parameter values $\varpropto = 0.00005480, \mu =0.01, \beta _h =0.04444, \beta _e =0.124, \alpha = 0.034, \sigma = 0.0006, \phi = 0.029, \nu = 0.02$ with the initial condition S(0) = 200, E(0) = 160, I(0) = 120 , B(0) = 130 and R(0) = 100 are used in the developed system. The dynamics of susceptible individuals, denoted as *S*, are illustrated in Fig. 3. After an initial sharp decline, the number of individuals rises and eventually stabilizes, reaching equilibrium respectively. The figures depict the dynamics of Exposed individuals (*E*), Infected individuals (*I*), and the presence of vibrio in the environment (*B*) in Figs. 8, 9 and 10 respectively. In each case, the population initially increases and, after a certain period, stabilizes respectively. The dynamics of Recovered individuals (*R*) are presented in Fig. 11, illustrating an initial rise, followed by a subsequent reduction and stabilization respectively.

This study forecasts future developments and suggests more effective ways to lessen the quantity of cholera disease that propagate through the intestine. When compared to traditional derivatives, the ABC technique produces better results for all sub-compartments at fractional derivatives. Also, it is asserted that the solutions for all compartments are more trustworthy and accurate when fractional values are reduced. The observation indicates an increase in cholera cases, whether individuals are symptomatic or asymptomatic. Additionally, it is noted that recovering rises by decreasing the fractional values as a result of adopting preventative and asymptomatic actions. It follows that by implementing both preventative and early detection strategies, we can manage cholera disease. It also forecasts what this research should find in the future and how we will be able to more effectively stop the spread of cholera disease infections throughout the population. Investigators may anticipate the future implications of this study.

## Conclusion

This paper presents a cholera disease model incorporating fractional-order dynamics, encompassing asymptomatic cases with and without medical treatment and Atangana–Toufik scheme is utilized to examine reliable findings. We provide guidance on how to stop the spread of illness by introducing the asymptomatic measures whose immune systems are strong without the need for medicine. The serious cholera disease is studied to determine its true global impact, both with and without symptomatic effects. For this purpose, the developed system is investigated quantitatively as well as qualitatively to verify its stable position for a continuous dynamical system and test the system with flip bifurcation analysis. So, we observe that the flip bifurcation does not exist in the developed system. Also the unique and bounded findings are verified for the developed fractional order system with the help of Banach space result to identify the bounded findings. Local stability analysis is conducted to ascertain the model’s behavior within bounded conditions, which is fundamental in understanding epidemic dynamics. The reproductive number $R_{0}$ is calculated to assess the rate at which the disease spreads within its sub-compartments, serving as a crucial parameter for identifying the potential for an outbreak in the community. Sensitivity analysis is applied to investigate the model’s response to variations in its parameters, providing insights into the factors that most significantly influence virus transmission. Solutions are derived for the developed system by using advanced tool Atangana–Toufik scheme. Also the error analysis has been made for the developed scheme to justify the accuracy of the obtained solution. We confirm the presence of the cholera disease with asymptomatic measures and analyze the effects of international efforts to stop its spread. It has been noted that early reduction of cholera disease is attributed to a robust immune system and combination treatment approaches. Using MATLAB, we conduct numerical simulations to observe the authentic dynamics of cholera disease control within the community, implementing a combination of symptomatic and asymptomatic measures to fortify the immune system. Also, numerical simulation is being utilized to test the true nature of the cholera influence in the community employing different fractional values, with the goal of developing control techniques to lower the risk factor of cholera in society. Predictions can also be generated based on validated results for further research, which will be beneficial in understanding the behavior and environmental propagation of the cholera disease as well as in the early detection procedure.

## Data Availability

The datasets used and/or analysed during the current study available from the corresponding author on reasonable request.
